# Dendritic distributions of *I*_h_ channels in experimentally-derived multi-compartment models of oriens-lacunosum/moleculare (O-LM) hippocampal interneurons

**DOI:** 10.3389/fnsyn.2015.00002

**Published:** 2015-02-27

**Authors:** Vladislav Sekulić, Tse-Chiang Chen, J. Josh Lawrence, Frances K. Skinner

**Affiliations:** ^1^Department of Fundamental Neurobiology, Toronto Western Research Institute, University Health NetworkToronto, ON, Canada; ^2^Department of Physiology, University of TorontoToronto, ON, Canada; ^3^Center for Structural and Functional Neuroscience, University of MontanaMissoula, MT, USA; ^4^Department of Biomedical and Pharmaceutical Sciences, University of MontanaMissoula, MT, USA; ^5^Department of Medicine (Neurology), University of TorontoToronto, ON, Canada

**Keywords:** h-channels, *I*_h_, dendrites, hippocampus, interneurons, multi-compartment model

## Abstract

The O-LM cell type mediates feedback inhibition onto hippocampal pyramidal cells and gates information flow in the CA1. Its functions depend on the presence of voltage-gated channels (VGCs), which affect its integrative properties and response to synaptic input. Given the challenges associated with determining densities and distributions of VGCs on interneuron dendrites, we take advantage of computational modeling to consider different possibilities. In this work, we focus on hyperpolarization-activated channels (h-channels) in O-LM cells. While h-channels are known to be present in O-LM cells, it is unknown whether they are present on their dendrites. In previous work, we used ensemble modeling techniques with experimental data to obtain insights into potentially important conductance balances. We found that the best O-LM models that included uniformly distributed h-channels in the dendrites could not fully capture the “sag” response. This led us to examine activation kinetics and non-uniform distributions of h-channels in the present work. In tuning our models, we found that different kinetics and non-uniform distributions could better reproduce experimental O-LM cell responses. In contrast to CA1 pyramidal cells where higher conductance densities of h-channels occur in more distal dendrites, decreasing conductance densities of h-channels away from the soma were observed in O-LM models. Via an illustrative scenario, we showed that having dendritic h-channels clearly speeds up back-propagating action potentials in O-LM cells, unlike when h-channels are present only in the soma. Although the present results were morphology-dependent, our work shows that it should be possible to determine the distributions and characteristics of O-LM cells with recordings and morphologies from the same cell. We hypothesize that h-channels are distributed in O-LM cell dendrites and endow them with particular synaptic integration properties that shape information flow in hippocampus.

## Introduction

Synaptic inputs arrive at the dendrites of the vast majority of neurons in the central nervous system. Dendrites often contain voltage-gated channels (VGCs), the density and distributions of which can critically impact synaptic integration and thus neuronal firing (Gulledge et al., 2005). While imaging and immunocytochemistry methods can provide qualitative information on the presence and location of channel subunits, the densities, distributions, and kinetic features of VGCs require electrophysiological measurements.

Dendritic recordings performed in pyramidal cells show an increasing density of hyperpolarization-activated channels (h-channels) in apical dendrites as a function of distance from the soma (Magee, 1998). Consistent with this distribution, immunocytochemical studies reveal an increasing density of the HCN1 subunit (one of the four known subunits of h-channels, HCN1-HCN4) toward distal locations of apical pyramidal cell dendrites in hippocampus, subiculum, and neocortex (Lörincz et al., 2002). This graded distribution allows the time course of excitatory synaptic events to be independent of dendritic input location (Magee and Cook, 2000; Williams and Stuart, 2000). A non-uniform h-channel distribution has also been shown to enable the selective propagation of synchronized frequencies across dendrites (Vaidya and Johnston, 2013). Such studies indicate that non-uniform distributions of h-channels in pyramidal cells can be of functional importance to hippocampal rhythmicity. Furthermore, h-channels can endow cells with a pacemaking role as well as setting their resting membrane potential (Biel et al., 2009).

While excitatory pyramidal cells make up 80–90% of the neuronal population, the smaller fraction of inhibitory cells, or interneurons, are critical controllers of network output and behavior (McBain and Fisahn, 2001; Klausberger and Somogyi, 2008; Kepecs and Fishell, 2014; Roux and Buzsáki, 2015). One interneuron type in the hippocampus, the oriens-lacunosum/moleculare (O-LM) cell located in the CA1 region, is of key interest because of its distinct morphology, specifically targeting distal regions of pyramidal cell dendrites. They also exhibit synaptic plasticity and have a demonstrated role in controlling information flow (Bartos et al., 2011; Leão et al., 2012). Interestingly, although O-LM cells are known to possess h-channels because of their “sag” response to hyperpolarizing currents (Maccaferri and McBain, 1996), the distribution of h-channels along the cell's membrane is at present unknown.

Given the extreme difficulty in performing dendritic recordings from interneurons in mice, we need multiple approaches to help determine and understand how the characteristics of VGCs on interneuron dendrites affect information processing. To date, there are only a few publications involving dendritic recordings in hippocampal interneurons (e.g., Martina et al., 2000; Hu et al., 2010). To address the specific question of whether h-channels may be present on dendrites, we developed and used an ensemble modeling approach (Sekulić et al., 2014), relying on our previously developed multi-compartment models of O-LM cells (Lawrence et al., 2006a). This computationally intensive approach involved close to two million simulations of multi-compartment O-LM models, an endeavor rendered feasible with the use of high performance computing (Loken et al., 2010) and automated extraction and analysis of electrophysiological features (Günay et al., 2009). Our ensemble modeling approach (Sekulić et al., 2014) allowed us to embrace the inherent biological variability that exists in neurons (Marder and Taylor, 2011), and we computationally examined whether any relationships between the various VGCs could be found, intentionally including dendritic h-channels for consideration. We found that only three (two involving h-channels uniformly located on dendrites and soma) out of more than 50 possible co-regulatory conductance balances exist in our models. Therefore, such large computational efforts using hippocampal interneurons can provide insight into neuronal properties. Moreover, the existence of co-regulatory balances to maintain output and function has been shown in biological neurons, in particular cells of the crab cardiac ganglion (Ransdell et al., 2013).

Our ensemble modeling investigations revealed the potential importance of dendritically located h-channels in maintaining cellular output (Sekulić et al., 2014). However, we found that even the best models in our ensemble were limited in their ability to capture the characteristic h-channel “sag” response. Therefore, in the present work, we examine how different h-channel activation kinetics and distributions affect model output. We carried out a full set of optimizations using output from two different O-LM cells and using two different model morphologies from previous studies. We found that many of the best fits were obtained with non-uniform distributions of h-channels along the dendrites. However, our results on whether the densities of h-channels were increasing or decreasing away from the soma were morphology-dependent. Our work indicates that with cell reconstructions and recordings from the same O-LM cells, it should be possible to reverse engineer our models to determine the distributions and characteristics of h-channels in biological O-LM cells. Further, we use models with different h-channel distributions to show that the speed of back-propagating action potentials is differentially affected. This suggests that dendritic h-channel distributions on O-LM cells play functionally important roles.

## Models and methods

**Figure 1:**
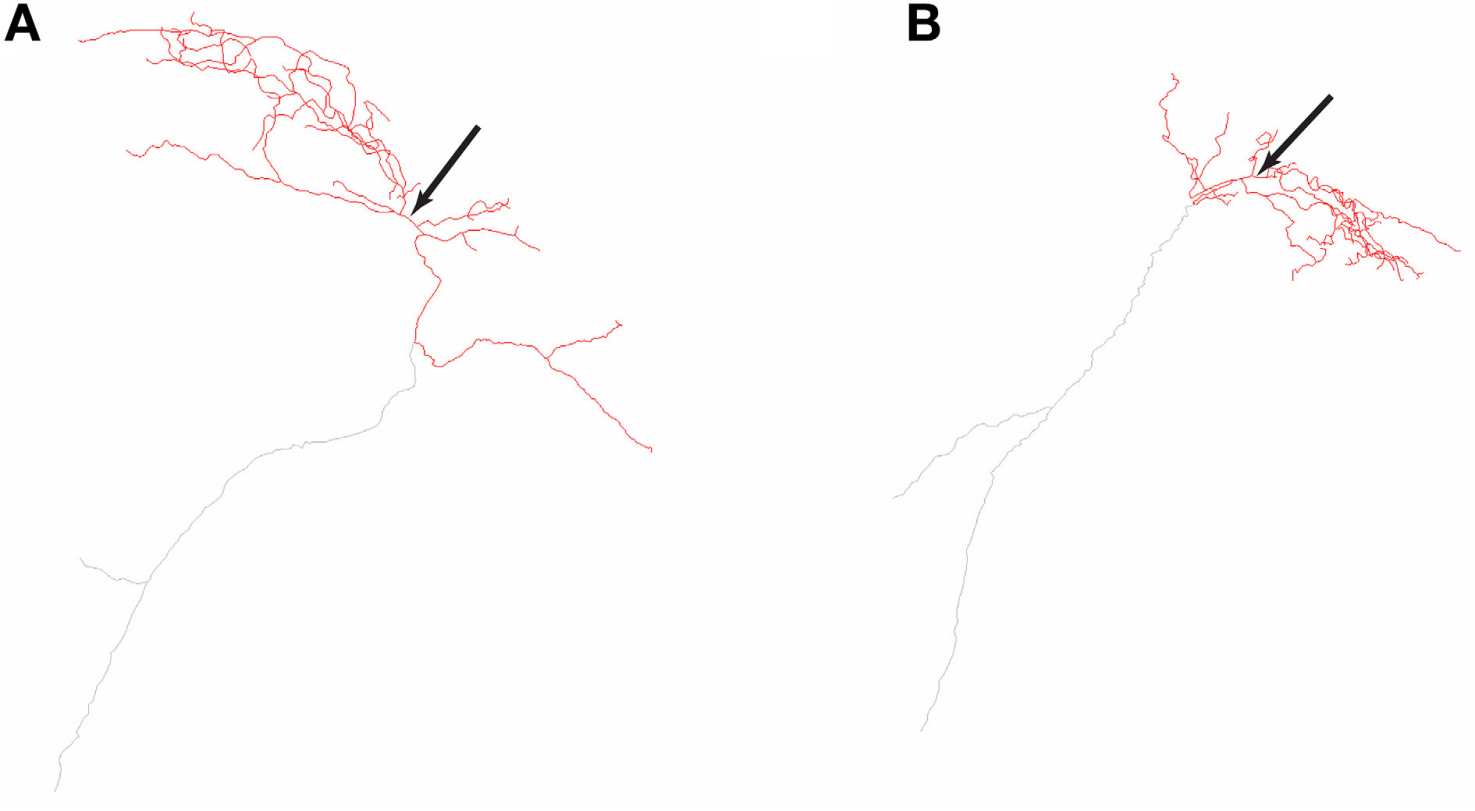
**Neurolucida reconstructions of the two O-LM cell morphologies used in this work**. Two-dimensional representations of O-LM cell morphological reconstructions originally obtained in Lawrence et al. (2006a) and used in this work for models R3, R4, referred to as morphology 1 **(A)** and S3, S4, referred to as morphology 2 **(B)**. Arrows denote location of cell somata. Dendrites are denoted in red, and truncated axons shown in gray.

The O-LM cell multi-compartment model description is provided in detail in Sekulić et al. (2014). It includes nine voltage-gated ionic currents: a transient sodium current, *I*_Na_ (with separately defined dendritic and somatic conductances, corresponding to *G*_Na,d_ and *G*_Na,s_, respectively), fast and slow delayed rectifier potassium currents, *I*_KDRf_ and *I*_KDRs_, respectively, a transient or A-type potassium current, *I*_A_, L- and T-type calcium currents, *I*_CaL_ and *I*_CaT_, respectively, a calcium-activated potassium current, *I*_AHP_, a hyperpolarization-activated mixed cation current, *I*_h_, and the Kv7/KCNQ/M current, *I*_M_. Most of the channels are distributed uniformly across both somatic and dendritic compartments, except for *I*_CaL_, *I*_CaT_, and *I*_AHP_, which are present only in the dendrites (Saraga et al., 2003). Unlike our previous work where we varied the location of *I*_h_ to be present either in the soma only or soma and dendrites in a uniformly distributed fashion, here we examined the case of somatodendritic *I*_h_ with non-uniform distributions. Two morphological reconstructions of O-LM cells were used, referred to as morphology 1 and 2 (Figure 1). Compartmentalization of the models done previously (Sekulić et al., 2014) resulted in the number of compartments for models of morphology 1 and 2 being 1291 and 2413, respectively.

In the previous work, the maximum conductance densities of the different ion channel currents were varied to generate a large database of models from which we extracted a subset that best captured the features exhibited in the biological O-LM cell recordings. From this subset of models, which we call highly-ranked models, we chose four for the present work. They are the first two highly-ranked models with morphologies 1 and 2 that have an *I*_h_ maximum conductance (*G*_h_) parameter value of 0.1 pS/μm^2,^ as this value is in the middle of the range of permissible *G*_h_ parameter values determined in our previous work (Sekulić et al., 2014). The parameters for the four chosen models, including maximum conductance densities for the various currents and passive properties, are shown in Table 1.

**Table 1:** **Baseline parameters for the models used in this work**.

**Parameter**	**Model R3**	**Model R4**	**Model S3**	**Model S4**
Morphology	Cell 1	Cell 1	Cell 2	Cell 2
*R*_m_ (Ω·cm^2^)	59,156	59,156	39,037.7	39,037.7
*C*_m_ (μF/cm^2^)	0.96857	0.96857	0.9	0.9
*I*_hold_ (pA)	−10.35	−11.39	−7.0	−6.9
*G*_Na,s_	107	60	220	107
*G*_Na,d_	230	230	230	230
*G*_KDRf_	506	506	506	506
*G*_KDRs_	42	42	42	42
*G*_A_	2.5	2.5	2.5	2.5
*G*_CaL_	25	25	50	12.5
*G*_CaT_	1.25	5	2.5	1.25
*G*_AHP_	5.5	2.75	2.75	2.75
*G*_h_	0.1	0.1	0.1	0.1
*G*_M_	0.75	0.375	0.375	0.75

*These include two passive properties, the specific membrane resistivity (R_m_) and specific membrane capacitance (C_m_), as well as the maximum conductance density values for the voltage-gated ion channels included in all four models. All maximum conductance density values (G_x_ for voltage-gated ionic current x) are specified in units of pS/μm^2^*.

### Ionic current model for hyperpolarization-activated channels (h-channels)

In the O-LM cell model, the conductance-based mathematical formulation used to represent current flow through h-channels is given by,

$$\begin{array}{l} \;I_{h}=G_{h}r\left(V-E_{h}\right),\\ \;\frac{dr}{dt}=\frac{\left(r_{\infty }-r\right)}{\tau }\\ r_{\infty }=\frac{1}{1+\exp\left(\frac{V-V_{1/2}}{-k}\right)} \end{array}$$

where *I*_h_ is the h-channel current, *G*_h_ is the maximum conductance density for the h-channels, *r* is the activation variable, *E_h_* is the h-channel reversal potential, r_∞_ is the steady state activation, *k* is the slope of activation, τ is the time constant of activation, *V*_1/2_ is the potential of half-maximal activation of *I*_h_, *V* is the membrane voltage, and *t* is time. The h-channel formulation here is taken from the previously developed reference O-LM models (Saraga et al., 2003; Lawrence et al., 2006a).

Three different sets of parameters based on experimental data have been used with various model equations of h-channels in O-LM cells in the literature: (*i*) Saraga et al. (2003) and Lawrence et al. (2006a), (*ii*) Kispersky et al. (2012), and (*iii*) Zemankovics et al. (2010). For (*i*) and (*ii*), O-LM cell data from Maccaferri and McBain (1996) were used along with non O-LM cell data and, for (*iii*), O-LM cell data was obtained as part of the same paper. Differences in the steady-state activation curves and reversal potentials exist in these models and are partly due to differences in junction potentials. The reversal potentials in the above papers are (*i*) *E_h_* = −32.9 mV, (*ii*) *E*_h_ = −20 mV, and (*iii*) *E*_h_ = −37.0 ± 5.9 mV. *V*_1/2_ in the h-channel models are (*i*) *V*_1/2_ = −80 mV, (*ii*) *V*_1/2_ = −75 mV, and (*iii*) *V*_1/2_ = −97.7 ± 5.0 mV. The value of *k* in the h-channel models are (*i*) −10 mV, (*ii*) −8 mV, and (*iii*) −8.9 ± 4.0 mV.

Although the differences in the reversal potentials and half-maximal activation of *I*_h_ affect the amplitudes of the hyperpolarization-induced sag, they cannot account for mismatches in the sag shape. These mismatches are likely due to activation time constant differences (See Figure 3 in Sekulić et al., 2014). As such, we chose to focus on the time constant equation aspects here. Although they have similar functional forms, the activation time constant for *I*_h_ differed substantially due to different parameter choices in previous studies. In particular, *I*_h_ models in (*i*) were based on using two data points from Maccaferri and McBain (1996) and (*ii*) did not base their time constant values on O-LM cell data. However, both (*i*) and (*ii*) had similar time constants as Zemankovics et al. (2010) at particular voltage values.

For (*i*), the *I*_h_ activation time constant was (in ms),

$$\tau =\frac{1}{e^{-17.9-0.116V}+e^{-1.84+0.09V}}+100$$

For (*ii*), the time constant used was (in ms),

$$\tau =\frac{150}{e^{25+0.5V}+e^{-\frac{6}{56}-\frac{1}{56}V}}+100$$

### Activation time constant formulation

The functional form of the model for the time constant of activation for *I*_h_, or τ (in ms), used in our optimizations is given by:

$$\tau =\frac{t_{1}}{t_{2}e^{t_{3}V+t_{4}}+t_{5}e^{t_{6}V+t_{7}}}+t_{8}$$

This is a generalization of the forms used in models of (*i*) and (*ii*) above, and has 8 parameters (*t*_1_, *t*_2_, …, *t*_8_). We chose this form as a simple generalization of that used previously to encompass the bell-shaped curve seen experimentally in h-channel activation time constants (Huguenard and McCormick, 1992). However, we note that in this formulation the eight parameters are not independent of each other. This is described in the Supplementary Material, as is the effect of each of the parameters on the asymmetrical shape of the bell-shaped curve.

In optimizing these parameter values, they were given the following constraints: *t*_1_ ≥ 0, *t*_2_ ≥ 0, *t*_3_ ≤ 0, *t*_4_ ≤ 0, *t*_5_ ≥ 0, *t*_6_ ≥ 0; *t*_7_, and *t*_8_ were allowed to take on any negative or positive value. Given this, the minimum value of τ is *t*_8_.

### Non-uniform distributions

**Figure 2:**
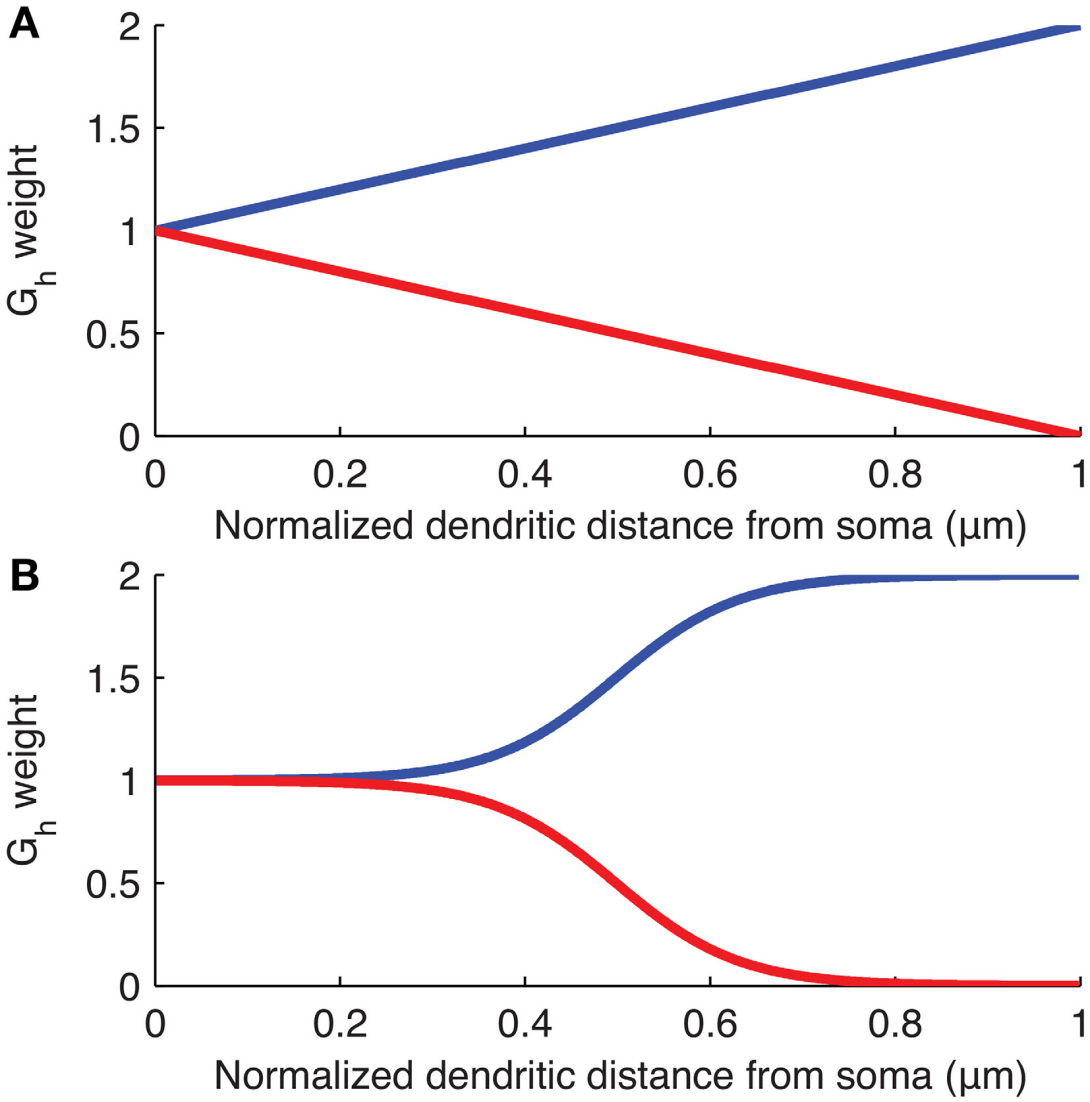
**Weighting of *G*_h_ as a function of distance along dendrites from soma**. Plots of the equations specifying the **(A)** linear and **(B)** sigmoidal distributions of *G*_h_ along the dendrites of the models used. The curves show linear or sigmoidal increase or decrease of *G*_h_ based on the dendritic scaling factor, *k*_d_, where *k*_d_ of 1 and −1 are plotted as, respectively, blue and red curves, in both **(A,B)**.

The distribution of *I*_h_ is varied in the computational model of the O-LM cell by modifying *G*_h_ at each compartment along the dendrites as a linear or sigmoidal function of the distance from that dendritic compartment to the soma (Figure 2). These distributions were chosen as the most reasonable to consider at this time given what is known about h-channel distributions in pyramidal cells (Magee, 1998; Golding et al., 2005). The linear distribution is defined by,

$$\;{\text{G}}_{h}\left(x\right)=G_{h}\left(d_{x}\frac{k_{d}}{D_{max}}+1\right)$$

The sigmoidal distribution is defined by,

$$G_{h}\left(x\right)=G_{h}\left(1+\frac{k_{d}}{1+e^{\left(\frac{\frac{1}{2}D_{max}-d_{x}}{20}\right)}}\right)$$

where *G*_h_(*x*) is the maximum conductance density assigned to dendritic compartment *x*, *G*_h_ is the baseline maximum conductance density derived from the original O-LM model in the database and is the value assigned to the somatic compartments (unless the baseline *G*_h_ itself was allowed to vary in the subsequent optimizations as a control case), *k*_d_ is the dendritic scaling factor, *D*_max_ is the maximum distance of any dendritic segment from the soma in microns, and *d*_x_ is the distance from the soma of the dendritic compartment *x*, in microns. Note that since an ion channel cannot have a negative conductance, if the evaluated *G*_h_(*x*) reaches zero for a particular scaling factor *k*_d_, then *G*_h_(*x*) is set to zero at all subsequent dendritic compartments distally from the soma so that there are no negative conductances defined for any dendritic compartment. This situation can occur in both the linear and sigmoidal distributions if *k*_d_ < −1 (See Figure 2 for plots of the linear and sigmoidal equations as a function of distance). In some of the simulations, the baseline *G*_h_ was allowed to vary (Sections “Optimal Fits are Obtained with Either Non-Uniform h-Channel Distributions and Fixed Baseline *G*_h_, or Uniform h-Channel Distributions and Fitted Baseline *G*_h_” and “Selection of Baseline *G*_h_ Values and Other Channel Conductances”).

### Experimental data

**Figure 3:**
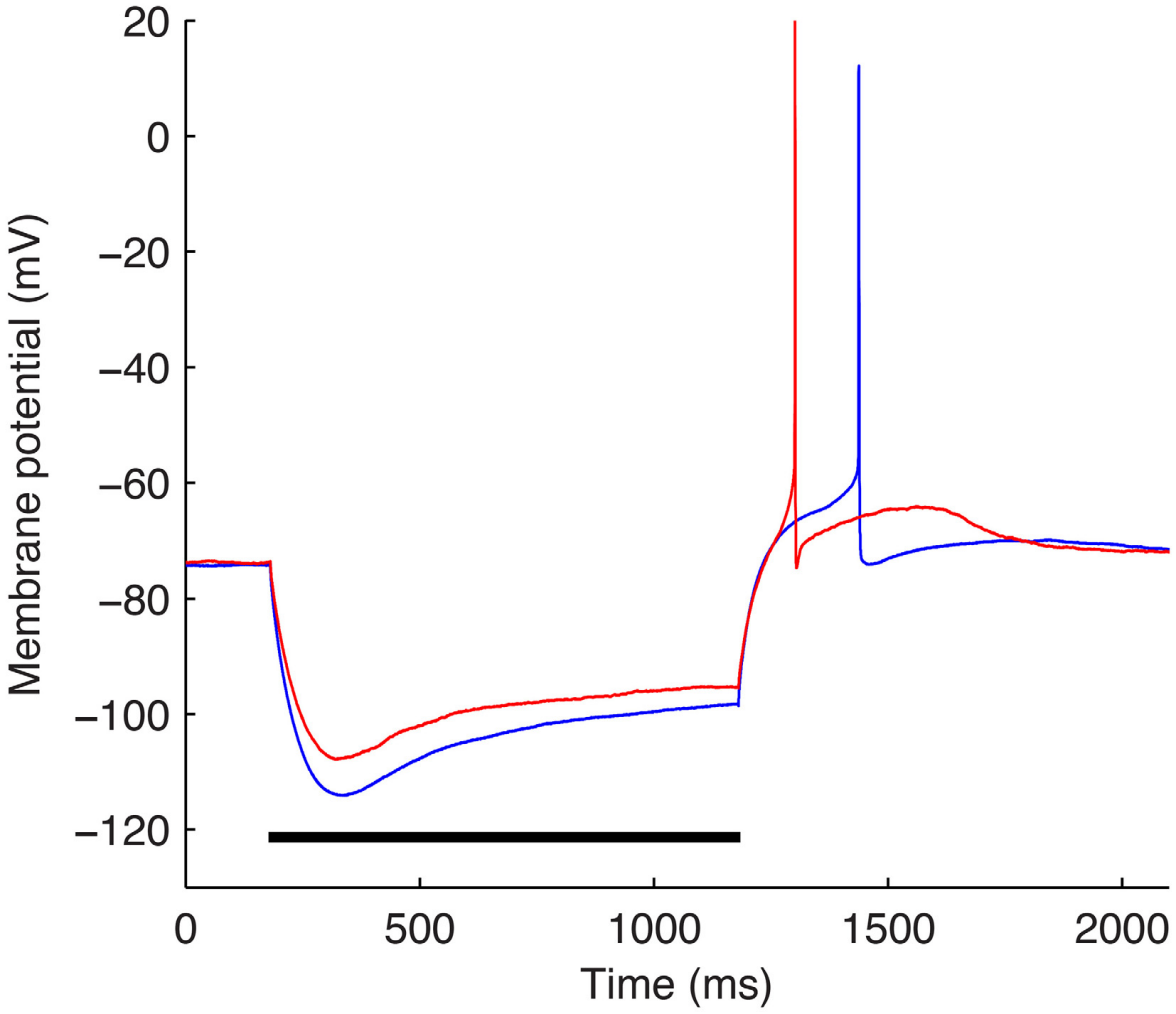
**The two experimental O-LM cell somatic membrane potential traces used in the optimization work**. Somatic membrane voltage response to a −90 pA hyperpolarizing current injection step for two experimental O-LM cells: cell 4525#4 (blue) and cell 4610#2 (red). Horizontal black bar shows time of application of −90 pA current injection step.

The optimizations were performed using a subset of the experimental data used in Sekulić et al. (2014) and described in detail in Lawrence et al. (2006b). Specifically, −90 pA somatic current steps of duration of 1 s from two different O-LM cells were used (Figure 3). The starting voltage before the hyperpolarizing step was −74 mV and for each optimization, the bias, or holding current required to set this voltage was specified within the optimization itself since the amount of current required varies depending on the passive properties and *G*_h_, which are parameters that were optimized as well.

### Modeling software and optimization

The model simulations and optimizations were performed in the NEURON simulation environment (Hines and Carnevale, 2001). The Multiple Run Fitter tool (MRF) within NEURON was utilized to optimize the various parameters in the models of this work. The MRF, in turn, uses the principal axis minimization procedure, or PRAXIS (Brent, 1973; Gegenfurtner, 1992). The principal axis method approximates the form of the local minimum of the desired function using a quadratic polynomial. The function to be minimized here was the sum of squared errors between the *V*_m_ somatic voltage values of the model and experimental trace the model was being fitted to, in units of mV^2^. To do this, the PRAXIS tool within the MRF was set to terminate when the local space around the minimum of the error function did not exceed a tolerance or difference of 1 × 10^−4^ mV^2^ of the error value, which is the default termination criterion specified by PRAXIS within NEURON. Nevertheless, multiple runs of PRAXIS could result in slightly smaller errors between model and experimental somatic voltage traces. Therefore, PRAXIS was run several times per model and experimental trace pair, each time taking the output (fitted parameter values) of the previous run as inputs to the next one, until the computed error did not change. We considered the parameter values obtained in this manner to be optimized and thus constituted the result of one optimization run.

Although models sometimes obtained different error values when performing identical optimization runs due to the PRAXIS algorithm converging at local minima, the overall results did not change across optimizations. The *n* values reported correspond to the number of optimization runs performed for each combination of experimental trace and morphology.

To evaluate whether non-uniform distributions are required to produce an optimal fit, we performed control simulations. They included cases with uniform dendritic *G*_h_, in which only the passive properties (*R*_m_, *C*_m_) or passive properties and *I*_h_ time constant (*t*_1_, *t*_2_, …, *t*_8_, *R*_m_, *C*_m_) parameters were fit. These are referred to as “Passive” and “Passive, τ” below. In the case of non-uniform dendritic *G*_h_, whether with linear or sigmoidal dendritic *G*_h_, all the passive properties and time constant parameters were fit as in the Passive, τ case, but also the dendritic scaling factor, *k*_d_, and holding current, *I*_hold_, were optimized. This was required as the somatic *V*_m_ of the models prior to the current injection step varied due to differing amounts of depolarization introduced by allowing non-uniformities of *G*_h_ in the dendritic compartments. In all cases, all parameters were optimized simultaneously. The sum of squared error between the model and experimental trace was computed with equal weighting across all portions of the trace. This included 180 ms of the trace prior to, and 120 ms after, the 1 s-long −90 pA hyperpolarizing current injection, a duration of 1.3 s in total. These two portions before and after the current injection period capture, respectively, the steady-state and depolarizing phases of *V*_m_.

When reporting optimization data, the values given correspond to the mean and standard deviation (SD) of the error value returned by PRAXIS at the end of the optimization runs described. Differences in populations of models are reported with *p*-values obtained by two-sample *t*-tests, with *p* < 0.05 indicating significant differences.

### Modeling inhibitory and excitatory postsynaptic currents onto O-LM cells

We modeled inhibitory postsynaptic currents (IPSCs) originating from parvalbumin-positive neurons of the medial septum-diagonal band of Broca (MS-DBB) using experimental data previously acquired (Garrett et al., 2013, 2014; Yi et al., submitted). The channelrhodopsin-2 variant ChETA was virally expressed in the MS-DBB of PV-CRE mice, using stereotaxic viral delivery methods similar to those described previously in hippocampus (Yi et al., 2015). IPSC photoactivation experiments were performed in transverse hippocampal slices to stimulate septohippocampal GABAergic afferents onto interneurons in the CA1 stratum oriens. A CsCl-based recording electrode was used to generate a large driving force (*V*_m_ = −60 mV, *E*_Cl_ = 0). The time course of decay of IPSCs mediated by parvalbumin-positive neurons of the MS-DBB averaged 8.5 ms. The average number of visualized release sites, determined from post-hoc analysis of ChETA-positive terminals adjacent to the somatodendritic domains of biocytin-filled cells, was 18 release sites.

We used model morphology 1 to insert putative MS-DBB GABAergic inhibitory conductances. The quantal amplitude of each inhibitory synapse was estimated from variance-mean analysis of the experimentally recorded somatic IPSCs. Given our model's resting potential (−74 mV) and *E*_Cl_ (−85 mV, Ferguson et al., 2013), this resulted in a postsynaptic inhibitory current of 6.5 pA. Eighteen inhibitory synapses were distributed randomly on the O-LM model morphology with a preference for perisomatic dendritic regions, based on preliminary experimental estimates (**Figure 10B**). The median distance of the inhibitory synaptic sites from the soma was 69.93 μm. When the synapses were activated, this resulted in a compound IPSP measured in the soma (**Figure 10C**). The inhibitory synapses were implemented in the model using the ExpSyn class in the NEURON simulation environment using the above parameters.

A single excitatory synapse, producing an excitatory post-synaptic current (EPSC), was modeled as the sum of rising (0.1 ms) and decaying (4 ms) exponentials, implemented by the ExpSyn2 class in the NEURON simulation environment. The single synapse was placed on a dendritic location 187.9 μm away from the soma, which was more distal to 17/18 of the IPSC location sites. The connection weight for the synapse was tuned so that a single somatic spike would be elicited upon activation of the synapse. See Carnevale and Hines (2006) for details on the ExpSyn and ExpSyn2 NEURON classes.

The simulation protocol started with 5 s of simulation time to achieve steady-state. At the 5 s mark, the IPSCs were simultaneously generated followed by a variable delay period, from 75 to 300 ms in duration, at which time the single EPSC was activated (**Figures 10C,D**).

## Results

Using two different O-LM cell morphologies and several ion channel conductance densities and distributions, we previously performed a model ensemble investigation with our developed multi-compartment models (Sekulić et al., 2014). This was done because the use of a single hand-tuned, canonical model to represent a neuron, no matter how detailed, is limited in the insight it can offer due to biological variability inherent even across cells of a single type (Marder and Taylor, 2011; Ransdell et al., 2013). From that study we obtained a population of models that captured electrophysiological features of O-LM cell recordings as exemplified by depolarizing and hyperpolarizing current steps. These so-called highly ranked models did not possess the same densities of conductances but instead exhibited balanced amounts in such a way that O-LM cell output was captured.

In the study here, we used four of these highly ranked models, two from each morphology, to perform a detailed examination of h-channels in O-LM cell models. Details of the models are given in the Methods and model parameter values are given in Table 1. We then used a subset of these models to illustrate differential back-propagating action potential responses given proximal, inhibitory inputs as might occur from the MS-DBB and a distal, excitatory input.

### Optimal fits are obtained with either non-uniform h-channel distributions and fixed baseline *G*_h_, or uniform h-channel distributions and fitted baseline *G*_h_

Since we are considering specific characteristics in a given cell type, we need to be clear about the data and model being used. Our experimental database from Sekulić et al. (2014) consisted of 10 cells with ± 90 pA steps. We selected two −90 pA step voltage recordings from two different cells, chosen intentionally since they exhibit different *V*_m_ minimum values. The somatic voltage traces for the two cells are shown in Figure 3.

We chose highly ranked models with a baseline *G*_h_ = 0.1 pS/μm^2^ in all four cases, so as to have consistent baseline values for comparison (See Table 1 for parameter values). We allowed the h-channel activation time constant to vary due to the paucity of data for activation time constant curves in the literature for O-LM cells (see Models and Methods). Also, since the experimental data were for a particular cell recording, the holding current—required to keep the model's initial *V*_m_ at −74 mV—the leak conductance, and specific capacitance were also fit for each optimization.

**Figure 4:**
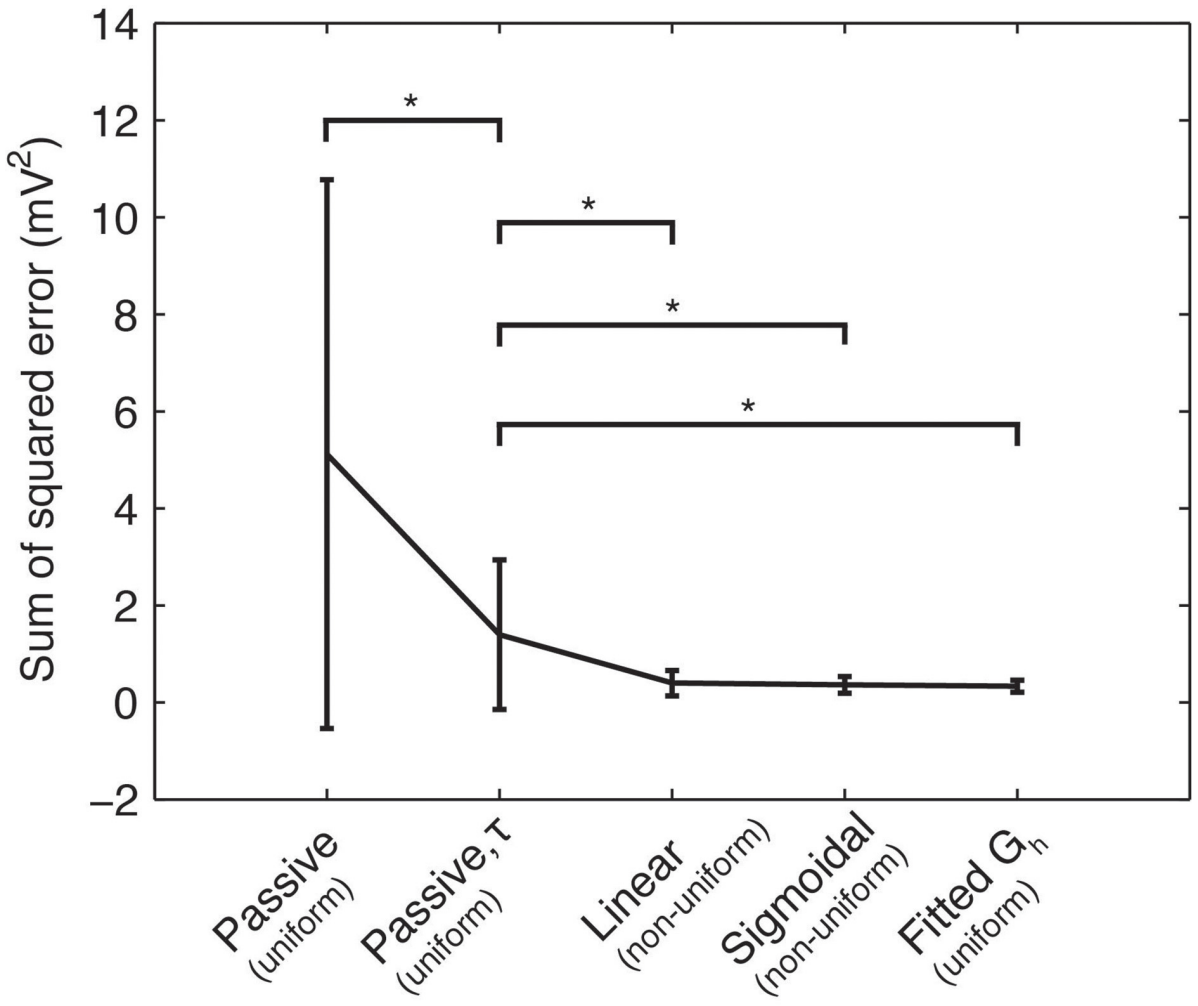
**Sum of squared errors between the model and experimental traces across different optimization procedures**. Means and standard deviations of sum-of-squared error measure (mV^2^) between model and experimental somatic membrane voltage response across four categories of model fitting: *R*_m_ and *C*_m_ only (“Passive”); *R*_m_, *C*_m_, and activation time constant for *I*_h_ (“Passive, τ”); *R*_m_, *C*_m_, τ, and linear dendritic *G*_h_ distribution (“Linear”); *R*_m_, *C*_m_, τ, and sigmoidal dendritic *G*_h_ distribution (“Sigmoidal”); *R*_m_, *C*_m_, τ, with *k*_d_ = 0 (resulting in a uniform dendritic *G*_h_ distribution), but the baseline *G*_h_ allowed to vary (“Fitted G_h_”). Single stars (“^*^”) denote significant differences in means across categories denoted, according to a two-sample *t*-test (*p* < 0.05). For clarity, the dendritic *G*_h_ distributions (uniform or non-uniform) are denoted in parentheses for each case.

Using the experimental recordings and the four model cells, we examined dendritic distributions of h-channels. We used either linear or sigmoidal non-uniform dendritic distributions with fixed baseline *G*_h_ (Figure 2). As control cases, we fitted the passive properties or the passive properties and activation time constant parameters (see Models and Methods). First, in comparing errors between the models with uniform distributions in which only the passive properties were fit (5.1165 ± 5.6543) and those in which the passive properties and the activation time constant were fit (1.3921 ± 1.5448), there was a large and statistically significant decrease in the error (*p* = 0.0385, Figure 4, compare “Passive” with “Passive, τ”). This is not too surprising as several more parameters (*t*_1_, *t*_2_, …, *t*_8_) were introduced in fitting the activation time constant. We obtained a further statistically significant decrease in the error when the dendritic scaling factor, or *k*_d_, was included in the optimizations, using either linear (0.3932 ± 0.2669, *p* = 0.0469, Figure 4, compare “Linear” with “Passive, τ”) or sigmoidal dendritic *G*_h_ distributions (0.3585 ± 0.1757, *p* = 0.0415, Figure 4, compare “Sigmoidal” with “Passive, τ”). However, there was no significant difference in the errors between models with linear and sigmoidal distributions (*p* = 0.7533, Figure 4, compare “Linear” with “Sigmoidal”).

In principle, one might expect that including this additional free parameter (*k*_d_) would reduce the error. However, we note that the equivalent case with no *k*_d_ parameter included is a possible outcome. That is, the case with fewer free parameters (“Passive, τ” in Figure 4) is a subset of the possible results for the optimization with more free parameters (“Linear” and “Sigmoidal” in Figure 4), specifically when *k*_d_ = 0. However, even the models with optimized *k*_d_ showed non-uniform distributions (*k*_d_ ≠ 0), although morphology-specific differences were apparent (see Section “Morphology-Specific Differences in Dendritic h-Channel Distributions and Total *G*_h_,” below). Further, if we allowed the baseline *G*_h_ to vary as well as *k*_d_, non-uniform distributions were still present (see Section “Selection of Baseline *G*_h_ Values and Other Channel Conductances,” below).

To explore the possibility that models with non-uniform distributions and fixed baseline *G*_h_ had lower errors than the control cases of fixed uniform distributions (the “Passive, τ” models in Figure 4) only because of having an extra free parameter, we performed additional simulations. These consisted of models where *k*_d_ was fixed to zero (corresponding to uniform distributions), and the baseline *G*_h_ itself was allowed to vary. This resulted in the same number of free parameters as the model optimizations with fixed baseline *G*_h_ but *k*_d_ having been allowed to vary. The resulting optimized *G*_h_ values, with corresponding model errors, are shown in Table 2. The fitted sum of square errors between the models and experimental data for the case with fitted baseline *G*_h_ were significantly smaller than the case with fitted passive properties and activation time constant (0.3293 ± 0.1252, *p* = 0.0366, Figure 4, compare “Passive, τ” with “Fitted *G*_h_”). Furthermore, there was no statistically significant difference in the errors for the fitted *G*_h_ case with those obtained for the models with non-uniform distributions and fixed baseline *G*_h_, for both linear (*p* = 0.4971) and sigmoidal (*p* = 0.7078) distributions (Figure 4, compare “Linear” and “Sigmoidal” with “Fitted *G*_h_”).

**Table 2:** **Models with fitted *G*_h_ and uniform dendritic *G*_h_ distributions**.

**Model**	**Experimental trace 1**		**Experimental trace 2**	
	**Fitted *G*_h_ (pS/μm^2^)**	**SSE**	**Fitted *G*_h_ (pS/μm^2^)**	**SSE**
R3 (morphology 1)	0.063403	0.2647	0.062724	0.35038
R4 (morphology 1)	0.070709	0.31578	0.091786	0.54136
S3 (morphology 2)	0.099042	0.24108	0.12114	0.35829
S4 (morphology 2)	0.1117	0.12841	0.11078	0.43418

*The G_h_ value for each model fitted by the optimization algorithm where k_d_ was set to zero. The sum of square error (SSE) between the model somatic voltage traces and corresponding experimental traces after fitting are shown*.

Therefore, the models in which the baseline *G*_h_ was allowed to vary but with uniform distributions (Figure 4, “Fitted *G*_h_”) can therefore be considered equally appropriate to models with fixed baseline *G*_h_ (where *G*_h_ = 0.1 pS/μm^2^) but non-uniform distributions (Figure 4, “Linear” and “Sigmoidal”). Thus, it seems unlikely that the models with non-uniform distributions of *G*_h_ had lower optimization errors compared to the control case (Figure 4, “Passive, τ”) simply due to the addition of a free parameter. Rather, the non-uniformity in the dendritic *G*_h_ distributions may be a possibility in biological O-LM cells.

**Figure 5:**
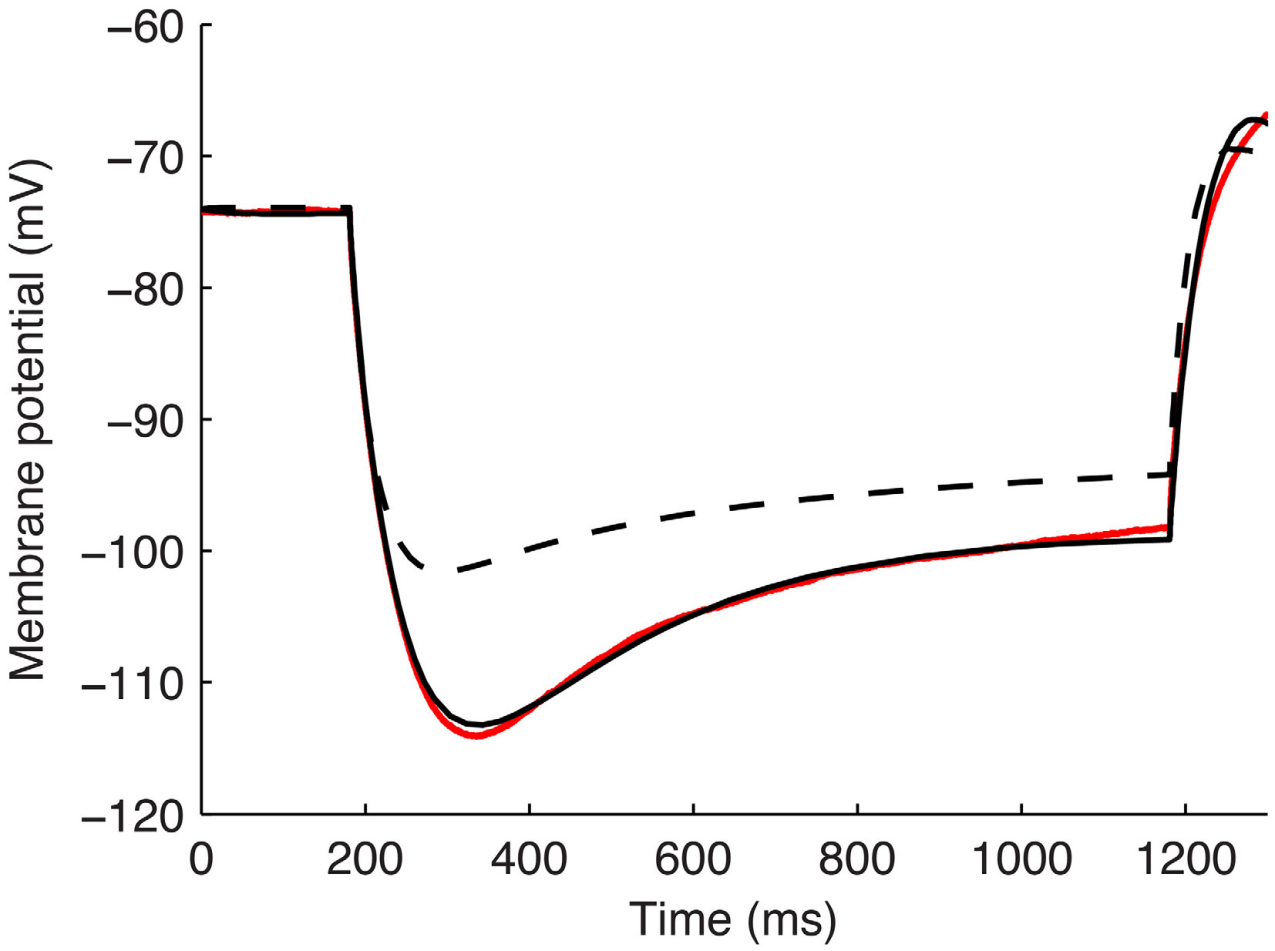
**Example model's somatic membrane potential before and after optimizing the passive properties and *I*_h_ characteristics**. Voltage traces showing experimental cell 4425#4 (solid red), model S3 with original parameters from highly-ranked database in Sekulić et al. (2014) (dashed black), and model S3 with optimized *R*_m_, *C*_m_, *I*_h_ activation time constant parameters *t*_1_, *t*_2_, …, *t*_8_, and dendritic scaling factor *k*_d_ for linear dendritic distribution of *G*_h_ (solid black).

An example of the improved fit between a model with the default parameters from the model database for *G*_h_ distributions and after fitting for kinetics, passive properties, and *k*_d_, is shown in Figure 5 (fitted *k*_d_ = −0.171783).

### Selection of baseline *G*_h_ values and other channel conductances

As noted in the Models and Methods, we used a particular value for the baseline *G*_h_ (0.1 pS/μm^2^) based on values obtained from our ensemble modeling work (Sekulić et al., 2014). We note this for two reasons. First, the exact value of *G*_h_ is likely not critical, but rather how it balances with other biophysical characteristics to maintain cellular output. However, the range of values *is* important. We think this because the range of appropriate *G*_h_ values found in our ensemble modeling study was at least an order of magnitude less than what has been measured in pyramidal cells (Sekulić et al., 2014). While these conductance measurements are variable, they have not been reported to be orders of magnitude different in variability. For example, Magee (1998) reported a conductance density range for h-channels of 1–10 pS/μm^2^ in CA1 pyramidal neurons. Second, these *G*_h_ values are dependent on balances with other conductances in the O-LM cell models (Sekulić et al., 2014). Since the recordings used did not specifically block other conductances, we also did not do so in our models. We now address these two aspects.

We optimized some of our models, allowing *G*_h_ in addition to *k*_d_ to vary in obtaining the best fit, using either linear or sigmoidal dendritic *G*_h_ distributions (errors for linear, 0.3446 ± 0.1338 and sigmoidal, 0.4842 ± 0.3116). There were no significant differences in the errors obtained when comparing the fits here with those when only *k*_d_ was varied, whether using linear or sigmoidal distributions (*p* = 0.7370 and *p* = 0.3836, respectively). In doing this, we found that the best fit only changed *G*_h_ by an amount less than an order of magnitude (Table 3). Note that 0.1 pS/μ m^2^ was the default *G*_h_ value for all four models used in this work, but when refit, there was never an order of magnitude change from this value, thus keeping the fitted values well outside of the range of conductance densities reported in CA1 pyramidal neurons.

**Table 3:** **Refitted *G*_h_ and *k*_d_ values in the models with both linear and sigmoidal dendritic *G*_h_ distributions**.

	**Model morphology 1 (R3), Experimental trace 1**	**Model morphology 1 (R3), Experimental trace 2**	**Model morphology 2 (S4), Experimental trace 1**	**Model morphology 2 (S4), Experimental trace 2**
**LINEAR DENDRITIC *G*_h_ DISTRIBUTION**				
*G*_h_ (pS/μm^2^)	0.0546	0.0467	0.2834	0.0655
*k*_d_	0.1870	1.036	−2.061	1.366
Total *G*_h_ (nS)	0.8373	0.9070	0.8518	0.8534
Model SSE	0.2538	0.4512	0.2063	0.4669
**SIGMOIDAL DENDRITIC *G*_h_ DISTRIBUTION**				
*G*_h_ (pS/μm^2^)	0.0933	0.0984	0.1308	0.1458
*k*_d_	−1.290	−0.2020	−0.5575	−0.1112
Total *G*_h_ (nS)	−0.2020	1.325	0.8734	1.189
Model SSE	0.2445	0.9019	0.2478	0.5426

*One model of each morphology was used in the re-fitting of G_h_ values, R3 for morphology 1, and S4 for morphology 2*.

We note that in the above Section, “Optimal Fits are Obtained with Either Non-Uniform h-Channel Distributions and Fixed Baseline *G*_h_, or Uniform h-Channel Distributions and Fitted Baseline *G*_h_,” we had examined how varying the baseline *G*_h_ with uniform distribution conditions would compare with non-uniform distributions and fixed baseline *G*_h_. Here, in doing these additional optimizations, we have expanded the possible cases by allowing both the baseline *G*_h_ and distributions to vary.

**Figure 6:**
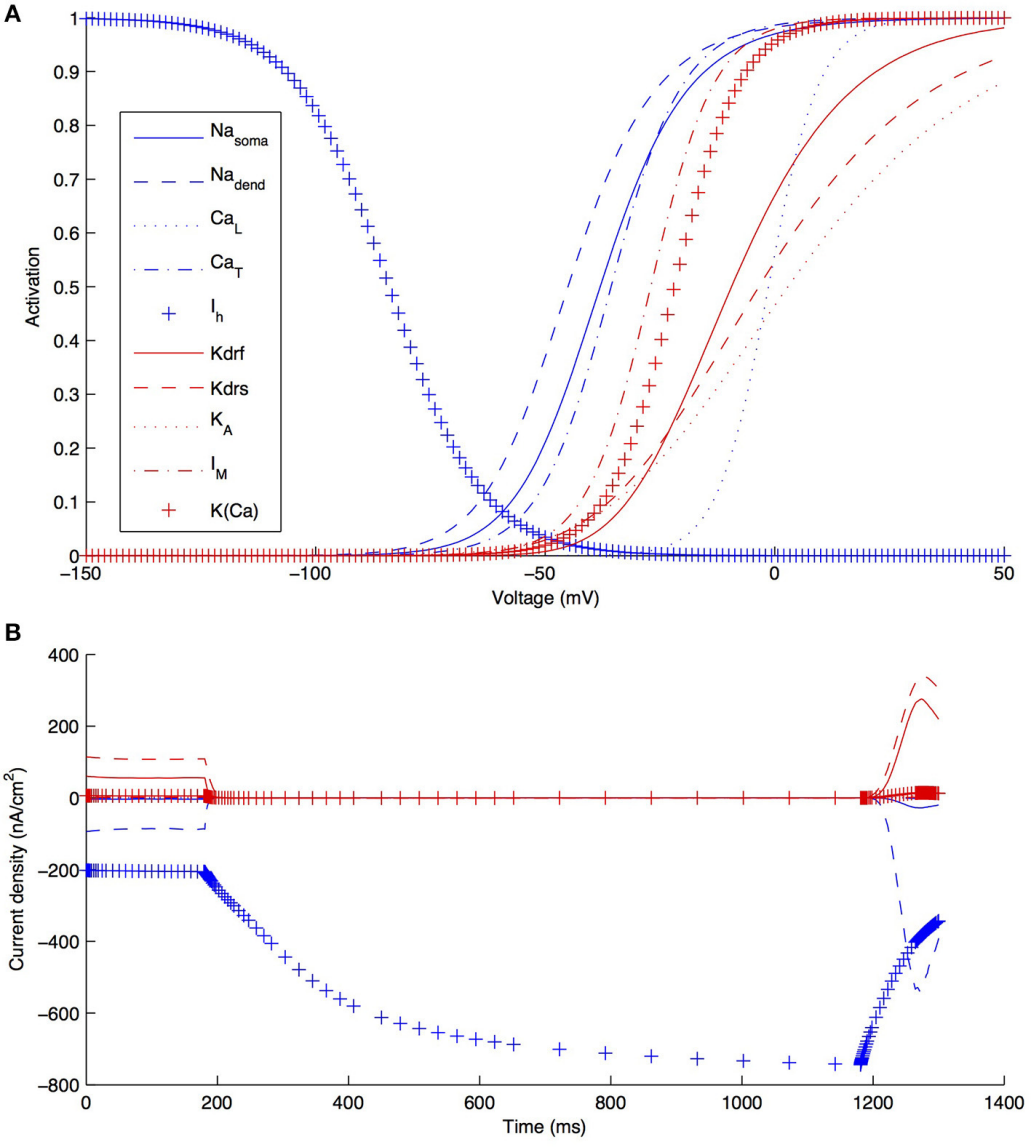
**Characteristics of all voltage-gated ion channels in the O-LM model**. **(A)** The voltage-dependence of steady-state activation for all ionic currents in the models; **(B)** current flows through the different ion channel types during a −90 pA hyperpolarizing current injection step in a dendritic compartment of model R3 adjacent to the soma.

**Figure 7:**
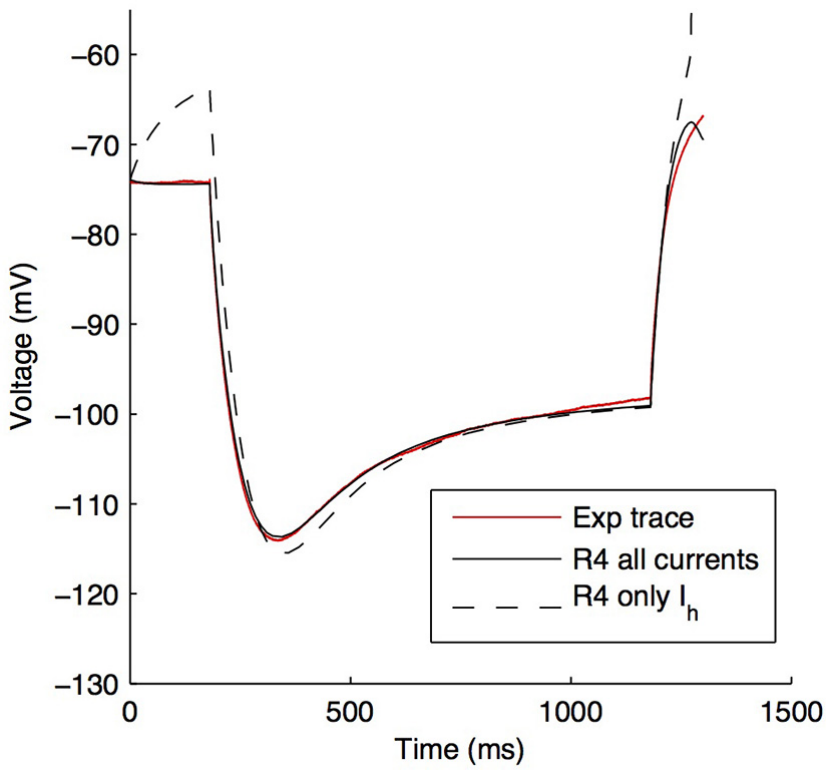
**O-LM model response to −90 pA hyperpolarizing current changes when all active voltage-gated channels, except *I*_h_, are blocked**. Somatic voltage traces in response to a −90 pA current injection step for experimental cell 4525#4 (solid red), optimized model R4 with all ion channels active (solid black) and optimized model R4 with only *I*_h_ active, with other channels blocked (dashed black).

Although other currents in O-LM cells were not blocked when applying the −90 pA current injection step, it is clear that other currents would have made some contribution, though it was expected to be minimal in the voltage range given by this current step regime. We demonstrate this by plotting the steady-state activation curves for all currents in the model (Figure 6A), which clearly shows that *I*_h_ is the predominantly active current in the voltage range present with −90pA steps. Furthermore, this is dynamically shown when plotting the current flows through the various voltage-dependent ion channels in the models (Figure 6B), where it is clear that the main active current during the 1 s-long duration of −90pA protocol was *I*_h_, starting from 181 ms into the virtual experiment. It is because of this that we were able to take advantage of the existing set of data to do the computational explorations of *I*_h_ distributions here. To illustrate this, we blocked all of the currents except for *I*_h_ in one of the models. As shown in Figure 7, this produced a small change only in the resulting somatic voltage trace with the −90 pA hyperpolarizing current injection protocol.

### Activation time constant characteristics for best fits

The optimization runs over the four highly-ranked models with linear and sigmoidal non-uniform *G*_h_ dendritic distributions resulted in a range of fitted parameters for the activation time constant of *I*_h_ (Table 4).

**Table 4:** **Fitted parameters for the activation time constant for *I*_h_**.

	**Original *I*_h_ model (Saraga et al. 2003)**	**Morphology 1, linear *G*_h_ distribution (n = 6)**	**Morphology 1, sigmoidal *G*_h_ distribution (n = 4)**	**Morphology 2, linear *G*_h_ distribution (n = 5)**	**Morphology 2, sigmoidal *G*_h_ distribution (n = 4)**
*t*_1_	1	0.1101±0.0771	0.3291±0.4531	0.0775±0.0824	0.2096±0.2290
*t*_2_	1	1.3034±0.5997	1.7319±0.8882	2.2111±2.1236	0.6100±0.8483
*t*_3_	−0.116	−0.0861±0.0154	−0.0969±0.0117	−0.0669±0.0365	−0.0871±0.0211
*t*_4_	−17.9	−17.9760±0.8265	−19.0218±2.1638	−17.6254±1.1320	−17.4178±1.9980
*t*_5_	1	1.1754±1.1460	1.0727±2.0780	7.6315±15.4502	0.3657±0.4023
*t*_6_	0.09	0.2566±0.1968	0.1719±0.1254	0.3850±0.3995	0.2499±0.2833
*t*_7_	−1.84	−5.1754±2.6165	−4.1309±1.9405	−4.5007±6.2876	−7.7548±4.9166
*t*_8_	100	106.3703±17.9600	93.4905±12.3784	108.2328±12.9816	109.9175±13.4007

*Means and standard deviations are shown separately for model fits of the two morphologies and across the two possible dendritic distributions of G_h_ (linear and sigmoidal)*.

**Figure 8:**
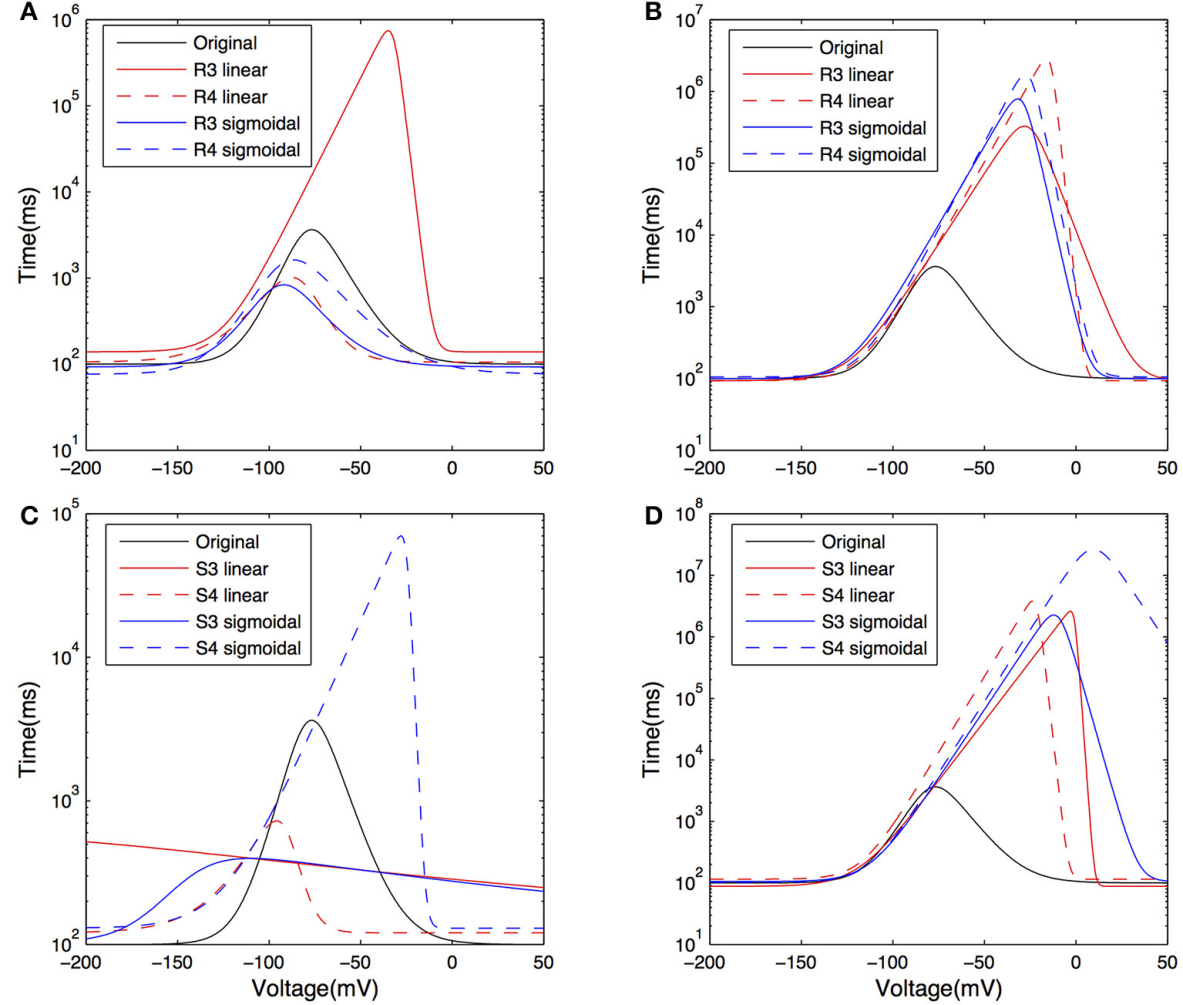
**Plots of the activation time constant curves of *I*_h_ for the models after optimization**. Curves showing the time constant of activation for *I*_h_, or τ±vs. voltage on a semi-log plot, for models with both morphologies and *R*_m_, *C*_m_, *t*_1_, *t*_2_, …, *t*_8_, and dendritic scaling factor *k*_d_ for dendritic distribution for *G*_h_ parameters optimized against both experimental traces. Models optimized against experimental trace 4525#4 with morphology 1 **(A)** and 2 **(C)**; models optimized against experimental trace 4610#2 with morphology 1 **(B)** and 2 **(D)**.

The activation time constants from the resulting fits are shown in Figure 8 on a semi-log plot. The original activation time constant used in Saraga et al. (2003) is also plotted for comparison purposes. We see that the activation time constant is similar to the original Saraga model for most of the portion of the membrane potential corresponding to when *I*_h_ is maximally active. This is from about −150 to −90 mV, as determined from *r*_∞_, the equation for the steady-state activation of *I*_h_ (see Models and Methods, also Figure 8A). Since the optimizations were done in these hyperpolarized ranges (Figure 3), this is somewhat expected. Otherwise, however, there is considerable variation. Taking into account the contributions of the various *t*_1_, *t*_2_, …, *t*_8_ parameters to the time constant (τ), it is also clear that the left hand side of the time constant curve is more constrained. That is, from Table 4, we note that *t*_3_, *t*_4_, and *t*_8_ had small variances relative to their mean values, whereas the other parameters had large variances. However, since only *t*_3_ and *t*_8_ can be considered as independent, we can mainly point to the (left hand side) horizontal scaling that seems to be a constrained aspect of the time constant curve. See Supplementary Material for more details. *t*_8_ represents the minimum τ-value, and is quite constrained. This suggests that the minimum time constant is relatively robust at about 100 ms.

### Morphology-specific differences in dendritic h-channel distributions and total *G*_h_

**Figure 9:**
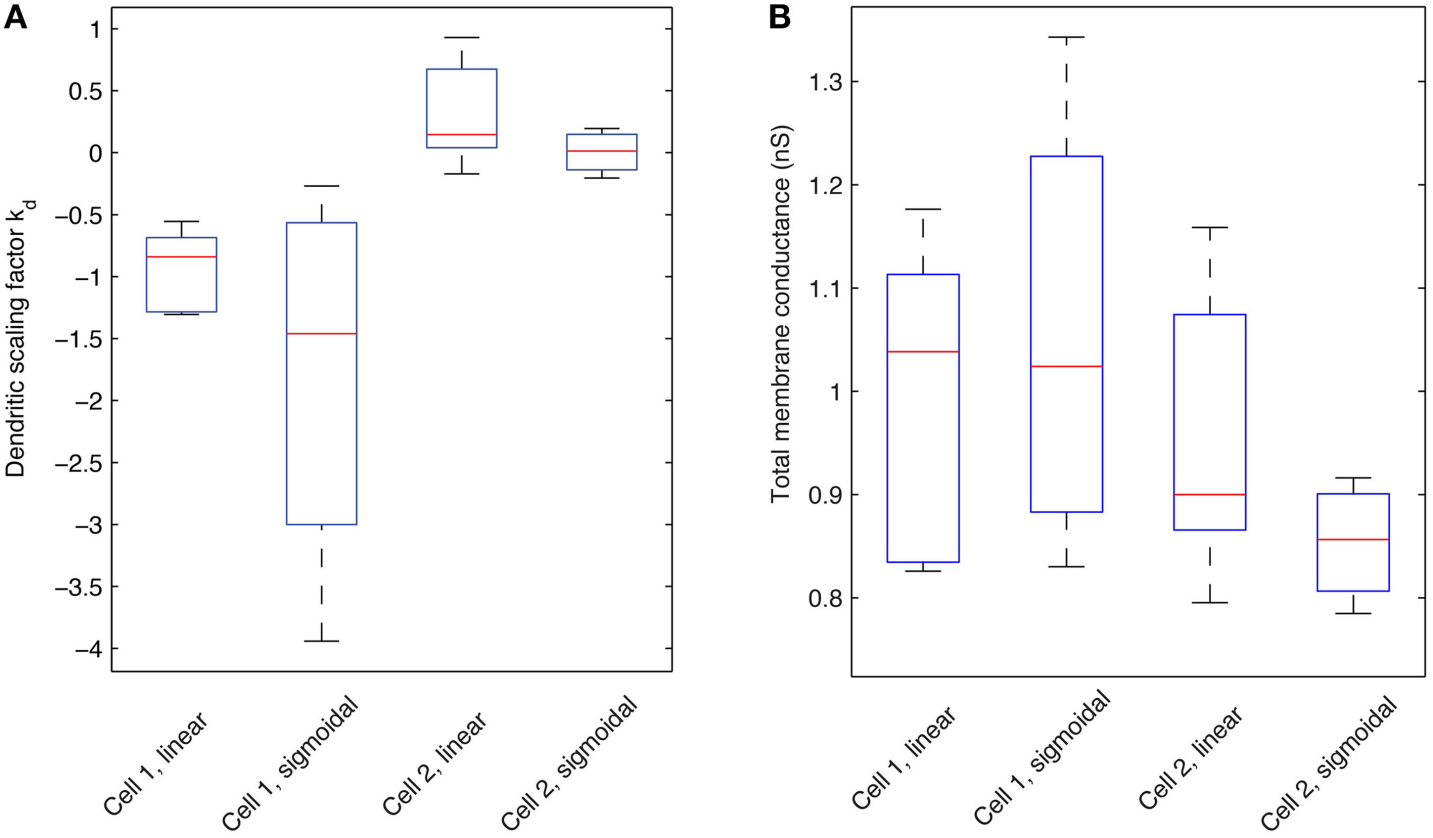
**Boxplots of the dendritic scaling factor *k*_d_ and total *G*_h_ membrane conductance across different model morphologies and dendritic *G*_h_ distributions**. **(A)** Statistics pertaining to the scaling factor, or *k*_d_, of models with optimized *R*_m_, *C*_m_, *t*_1_, *t*_2_, …, *t*_8_, and dendritic scaling factor *k*_d_ for both linear and sigmoidal dendritic distribution for *G*_h_ against both experimental traces used. Red lines indicate the median; box edges denote the 25th and 75th percentiles; whiskers extend to the extremes of the *k*_d_ values for each category. **(B)** Statistics pertaining to the total *G*_h_ membrane conductance (in nS) of models with optimized *R*_m_, *C*_m_, *t*_1_, *t*_2_, …, *t*_8_, and dendritic scaling factor *k*_d_ for both linear and sigmoidal dendritic distribution for *G*_h_ against both experimental traces used. Red lines indicate the median; box edges denote the 25th and 75th percentiles; whiskers extend to the extremes of the total *G*_h_ values for each category.

We next addressed the distributions of h-channels in our models by examining the fitted dendritic scaling factors, or *k*_d_ values, for the models of both morphologies and non-uniform dendritic distributions—linear or sigmoidal—and fixed baseline *G*_h_ (Figure 9A). The medians of the *k*_d_ values were negative for models with morphology 1 (−0.8409 for linear, *n* = 6, and −1.4605 for sigmoidal distributions, *n* = 4) and positive for models with morphology 2 (0.1454 for linear, *n* = 5, and 0.0138 for sigmoidal distributions, *n* = 4), using both experimental traces in the optimizations in each case.

Since the equations of location-dependent *G*_h_(x) with *k*_d_ = 0 would result in *G*_h_(x) resolving to *G*_h_ for all dendritic compartments *x*, a *k*_d_ of zero is equivalent to the case of uniform dendritic distributions of *G*_h_. Assuming that *k*_d_ values resulting in greater than ± 10% of the baseline, or somatic, *G*_h_ value in the most distal dendrites of O-LM models represent non-uniform distributions, then 10/10 of fitted models with morphology 1 and 6/9 of fitted models with morphology 2, across both linear and sigmoidal dendritic *G*_h_ distributions, were non-uniform. Thus, in our examinations with a fixed baseline *G*_h_, non-uniform distributions of *I*_h_ in the dendrites were present in the majority of our O-LM models.

Given that different distributions were obtained for the two different morphologies, we examined why this might be the case. In particular, all models with morphology 1 possessed negative *k*_d_ values, representing decreasing densities of h-channels moving away from the soma along the dendrites, whereas for models with morphology 2, *k*_d_ was either slightly negative or positive. This means that whether the h-channel densities decrease or increase away from the soma is morphology dependent. Considering this, we first note that there was consistency in the specific membrane resistivity, *R*_m_ (or inverse leak), and specific membrane capacitance, *C*_m_, values encompassed in the fits for each morphology (Table 5).

**Table 5:** **Fitted specific membrane resistivity and capacitance values in the models**.

**Model**	**Linear dendritic *G*_h_ distribution**		**Sigmoidal dendritic *G*_h_ distribution**	
	**Morphology 1 (n = 6)**	**Morphology 2 (n = 4)**	**Morphology 1 (n = 5)**	**Morphology 2 (n = 4)**
*R*_m_ (Ω . cm^2^)	116,300 ± 9,103.88	64,364.7 ± 7,320.86	117,185 ± 9,963.84	62,065.2 ± 4,620.38
*C*_m_ (μF/cm^2^)	0.7084 ± 0.0744	0.7113 ± 0.0747	1.2001 ± 0.1556	1.2094 ± 0.1448

*The means and standard deviations of the specific membrane resistivity (R_m_) and specific membrane capacitance (C_m_), grouped according to model morphology and dendritic G_h_ distribution (linear or sigmoidal)*.

These results are reasonable given that the two morphologies differed in key features such as dendritic extent (see Figure 1—note that the full axonal tree is not represented in the models). The surface area for morphology 1 is 16,193.6 μm^2^ and 9980.1 μm^2^ for morphology 2, and the maximum length of the furthest dendritic compartment is 308.92 μm for morphology 1 and 193.1 μm for morphology 2. These differences can account for why a cell with a larger surface area would be fit with a smaller leak conductance density (represented by *R*_m_) for a given experimental recording. Thus, even though the recordings from two different cells did not have identical passive properties (see Figure 3), the morphology differences were more critical here.

We next considered what the total h-channel conductance would be in each cell morphology given the different distributions, and noted that there was also consistency in the total *G*_h_ conductance in a morphology-dependent manner that was maintained regardless of the distribution of dendritic *G*_h_—linear or sigmoidal (Figure 9B). The median of the total *G*_h_ conductance for models with morphology 1 was 1.0381 and 1.0240 nS for the linear and sigmoidal case, respectively, and the total *G*_h_ conductance was 0.8998 and 0.8564 nS for the linear and sigmoidal case, respectively, for models with morphology 2. For comparison purposes, the total *G*_h_ conductance for models with uniform *G*_h_ dendritic distributions, that is, the original models extracted from the database in Sekulić et al. (2014), is 1.4435 nS for morphology 1 and 0.8518 for morphology 2. This result, together with the per-morphology characteristics above, suggest that there is a morphology dependent amount of required total *G*_h_ when fits are obtained. This helps explain why different non-uniform distributions may have been obtained for the two different morphologies since by variously having increasing or decreasing densities of dendritic h-channels, a total *G*_h_ conductance was maintained in a given morphology. As such, it is essential to have morphology specific recordings in determining distributions, as well as constraints on *G*_h_. A balance with other active conductances would also be a factor.

### Back-propagating action potential speeds depend on dendritic h-channel distributions

**Figure 10:**
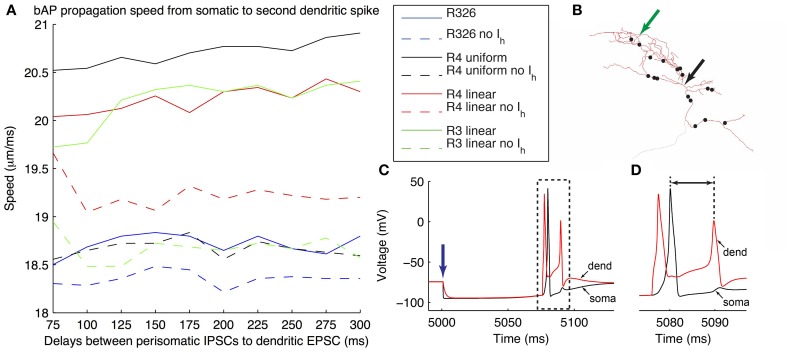
**Illustration of generation of back-propagating action potentials in O-LM model in response to inhibitory MS-DBB-like inputs and excitatory inputs**. **(A)** Speed of back-propagating action potential (bAP) measured in the model in response to different delays between the initial inhibitory post-synaptic current activation (IPSCs) and subsequent excitatory post-synaptic current activation (EPSCs). Several models are shown, including somatic *I*_h_ only (R326). In all cases, bAP speeds are also shown in the case with *I*_h_ currents blocked. **(B)** Locations of MS-DBB-like IPSC inputs used on the models with morphology 1 (black dots). Black arrow denotes location of soma; green arrow denotes location of the injected EPSC. **(C)** Representative superimposed somatic and dendritic *V*_m_ responses during the applied protocol; blue arrow denotes time at which the initial IPSC was evoked. The model shown is R4 linear, with IPSC-EPSC delay of 75 ms. **(D)** Expanded view of traces corresponding to the dashed box in **(C)** showing the bAP measured, from the soma to the second dendritic spike (black line with arrows). The y-axis is shared with **(C)**.

To investigate how different dendritic distributions of *I*_h_ on O-LM cells might be important, we need to examine the effects of dendritic *I*_h_ on synaptic integration. To do this, we considered an illustrative scenario of back-propagating action potentials (bAPs). Extra-hippocampal structures such as the MS-DBB provide inhibitory drive to hippocampal interneurons, thus disinhibiting dendritic regions of hippocampal pyramidal cells, at theta frequencies (Gulyás et al., 1990; Vertes and Kocsis, 1997; Hangya et al., 2009). Moreover, hippocampal O-LM cells are specific targets of parvalbumin-positive (PV^+^) cells from the MS-DBB (Chamberland et al., 2010). This raises the question of whether interactions between theta frequency-timed inhibitory post-synaptic currents (IPSCs) from MS-DBB PV^+^ cells interact with local *I*_h_ currents in O-LM cells to support theta-patterned output. As precedent, *I*_h_ has been shown to interact with inhibitory inputs onto subthalamic nucleus (STN) neurons to reduce burst firing and promote single post-inhibitory rebound spikes (Atherton et al., 2010). Moreover, O-LM cells have highly active dendrites and could support back-propagating action potentials (bAPs). O-LM cells have been shown to be capable of supporting long-term potentiation (LTP), which may arise from Hebbian synaptic plasticity rules, dependent on the coincident timing of back-propagating spikes and synaptic signals (Perez et al., 2001; Bartos et al., 2011). We therefore examined how IPSCs onto O-LM cells from the MS-DBB followed by a single EPSC would evoke bAPs in O-LM models. Previous work has indicated that the spatial location of synaptic input from medial septal PV^+^ cells onto O-LM cells in CA1 seems to be predominantly localized to the perisomatic region (Garrett et al., 2013, 2014; Yi et al., submitted). We arranged putative MS-DBB inhibitory synaptic sites onto model morphology 1 (Figure 10B; see Models and Methods). Representative models of morphology 1 with uniform and linear distributions of dendritic *I*_h_, as well as a model from the original database with only somatic *I*_h_ – that is, no dendritic *I*_h_ – were used. In the cases of the model from the database (R326) and the model with uniform dendritic *G*_h_ (R4, uniform), the passive properties and bias current were optimized to one of the experimental traces. This was done to allow a valid comparison with the other two models (R4, linear and R3, linear), that already had these parameter values fit to the experimental data (see Table 6 for the fitted parameter values for these models).

**Table 6:** **Parameter values for the O-LM models used for EPSC and IPSC stimulation**.

**Parameter**	**R326, no dendritic *G*_h_**	**R4, uniform dendritic *G*_h_**	**R4, linear dendritic *G*_h_**	**R3, linear dendritic *G*_h_**
*R*_m_ (Ω . cm^2^)	81,355	121,850	123,540	124,200
*C*_m_ (μF/cm^2^)	0.5	0.62	0.64	0.63
*I*_hold_ (pA)	−6.74	−11.39	+7.42	−5.93
*k*_d_	N/A	0	−1.3	−0.685

To simplify matters in this illustrative examination we simultaneously activated all inhibitory synapses. This was done at various delays after the evocation of a single dendritic EPSC. Simulations were performed for the models with *I*_h_ and with *I*_h_ blocked (by setting the maximal conductance to zero), in order to allow comparisons of the effects of having h-current in dendrites or soma on the synaptic inputs to be made within a model. We then determined back-propagating action potential speeds by measuring the distance between soma and dendritic recording location in the model divided by the difference in peak times between the somatic and dendritically-measured spikes (Figure 10D, black line with arrows). The results showed that when the h-current was blocked, models with dendritic *G*_h_ showed a clear decrease in bAP speeds across all EPSC-IPSC delays. In contrast, the model with somatic *G*_h_ only showed a minimal decrease in bAP speeds (Figure 10A—compare solid versus dashed lines for all models). The slowdown in bAP speeds when h-currents were blocked likely involves the reduced membrane depolarization due to the lack of inward h-current. This, in turn, would result in a later time for the subsequent dendritic spike. Given the link between bAPs and synaptic plasticity, this illustration suggests a functional consequence of dendritic *I*_h_.

## Summary and discussion

In this work we honed in specifically on the question of the h-channel representation, namely kinetics, conductance densities, and dendritic distributions, in multi-compartment models of O-LM cells for two reasons. First, our previous ensemble modeling work showed that even the best O-LM models lacked appropriate “sag” responses, compared to experimental data (Sekulić et al., 2014), thus showing that it was important to re-examine the *I*_h_ channel model itself. Second, although it is well documented that *I*_h_ exists on the dendrites of pyramidal cells (Magee, 1998), it is not known whether and in what fashion they are distributed on O-LM cell dendrites. The presence or absence of dendritic *I*_h_ could greatly affect synaptic integration in O-LM cells, especially as diverse excitatory (from local CA1 pyramidal cells) and inhibitory (from septohippocampal and local GABAergic sources) synapses onto CA1 O-LM cells have been characterized (Ibáñez and Freund, 1995; Freund and Antal, 1988; Tyan et al., 2014). We specifically illustrated the effect on back-propagating action potential (bAP) speeds for somatic only, uniform somatodendritic and non-uniform somatodendritic distributions of h-channels in O-LM cells (Figure 10). We found that the presence of h-channels in somatic locations minimally affects bAP speeds, whereas with dendritic h-channels, bAP speeds are decreased. It is interesting to note that in a small rhythmic neural network system, it has been found that modulation of *I*_h_ could control synaptic strength, with the expression of *I*_h_ being co-localized with synaptic locations (Goeritz et al., 2011).

We used four highly-ranked O-LM multi-compartment models from our previous work, which included two different morphologies, and fitted the *I*_h_ model parameters to two specific experimental cell recordings taken from O-LM cells in mice using a −90 pA current clamp experimental protocol. We found that the optimized parameters across all models included non-uniform distributions of *G*_h_. Most models expressed decreased densities of *G*_h_ further from the soma. However, some models showed increased densities, and uniform dendritic densities were also possible. We found that these differences in distributions of dendritic *G*_h_ depended on a combination of factors including model morphology and total *G*_h_ conductance, indicating that different models can appropriately match O-LM cell output in the hyperpolarized regime. We note that the result that non-uniform distributions possibly expressed in O-LM cells cannot be disregarded simply due to the number of free parameters. At present, it is not possible to conclude one way or another whether *G*_h_ distributions are likely to be uniform or non-uniform in O-LM cell dendrites. However, what our work clearly shows is that a range of *G*_h_ values and dendritic distributions allow us to obtain models of O-LM cells that fit experimental traces very well. Because both of these parameters are unconstrained at present by experimental data from O-LM cells, we expect that with experimental data pertaining to one of these parameters, it will be possible to use the approach in this work to predict the other parameter.

Our results demonstrate that it is critical to match cell morphologies with the electrophysiological recordings used. While this may seem obvious, it is not obvious that one would consistently get particular distributions, as we did using morphology 1, where we found that models with that morphology always had decreasing densities of *G*_h_ away from the soma, given a fixed baseline *G*_h_. As such, our computational modeling work here indicates that with multiple, matched O-LM cell morphologies and recordings— that include measures of *G*_h_ conductance—we can predict what h-channel distributions are likely to be present on O-LM cells. We note that here, the O-LM morphologies used in our models were obtained from earlier studies (Lawrence et al., 2006a) and did not correspond to the same O-LM cells from which the experimental recordings were taken (Lawrence et al., 2006b). We further note that the recordings used were non-dendritic, as dendritic recordings are highly challenging to obtain. Additional model complexities such as non-uniform passive properties (e.g., see Nörenberg et al., 2010), different sigmoidal fits (Golding et al., 2005), and different activation curves and time constants at different dendritic locations may also exist, and with more experimental constraints, these aspects could be examined in subsequent investigations.

To directly show the existence of h-channels in dendrites, immunohistochemical labeling studies could be done. To date, immunohistochemical labeling has shown that O-LM cells may express the HCN1, HCN2, and HCN4 subtypes (Notomi and Shigemoto, 2004; Matt et al., 2011; Hughes et al., 2013), although these studies did not specifically examine interneuron dendrites for the presence of HCN subtypes, and were performed in rat, whereas the experimental data and modeling used in our work pertain to mouse O-LM cells. The variety of HCN subtypes shown to be present in oriens-alveus (O-A) and specifically O-LM cells, in conjunction with heterogeneous co-expression of different HCN subtypes in subcellular compartment-specific manner in thalamic reticular neurons (Abbas et al., 2006), raises the possibility of a complex picture emerging regarding differential contribution of different subtypes of HCN channels in a location-specific manner in O-LM cells. This is corroborated by kinetic studies that show O-LM cells expressing fast- and slow- components of *I*_h_ in the range of both HCN2 and HCN4 subtypes (Santoro et al., 2000). A further implication is the possibility of heteromeric *I*_h_ channels formed by multiple HCN subtypes; Chen et al. (2001), for example, demonstrated mixed kinetics of *I*_h_ when HCN1/2 heteromers were co-expressed in Xenopus oocytes. Due to these complexities of *I*_h_ channel expression in O-LM and other cell types, future modeling work could assist in evaluating the consequences of mixed HCN subtype expression in terms of *I*_h_ kinetics or, conversely, help constrain or corroborate immunohistochemical labeling by matching the *I*_h_ kinetics demonstrable in the models with the kinetics of the appropriate HCN subtypes being examined in physiological O-LM cells.

The functional contributions of dendritic h-channels in O-LM cells, whether of uniform or non-uniform distributions, is yet to be elucidated. Our results with inhibitory MS-DBB proximal synaptic inputs, in conjunction with a distal excitatory spike-generating synaptic input, show that the presence of dendritic h-channels could substantially change the effect of synaptic integration. We demonstrated this by the measure of back-propagating action potential (bAP) speeds. Blocking the h-currents in the models with somatic h-channels hardly affected bAP speeds, whereas in models with dendritic h-channels (with either uniform or non-uniform dendritic distributions), blocking h-currents resulted in a clear decrease of bAP speeds. This differential effect on bAP speed depending on the presence of dendritic h-channels may have implications for LTP as Hebbian plasticity rules are intimately dependent on small time differences between incoming presynaptic and back-propagating spikes (Perez et al., 2001; Bartos et al., 2011). However, our example is illustrative to demonstrate that dendritic h-channels can have functional consequences.

In conclusion, our modeling work strongly suggests that h-channels are present on dendrites of O-LM cells. Furthermore, with morphology and experimental recordings obtained from the same cell we expect to be able to determine the distributions of h-channels in O-LM cells. To minimize interference from other voltage-dependent conductances, these recordings should be done with all other currents blocked during the hyperpolarizing steps. Having multiple hyperpolarizing steps as well as recordings with the h-channels blocked, with the same cell morphology, would allow *G*_h_ to be constrained for the particular O-LM cell, and we would expect this value to lie within the range determined from our previous ensemble modeling study (Sekulić et al., 2014).

### Conflict of interest statement

The authors declare that the research was conducted in the absence of any commercial or financial relationships that could be construed as a potential conflict of interest.
