# Supplementary material for: Dendritic distributions of Ih channels in experimentally-derived multi-compartment models of oriens-lacunosum/moleculare (O-LM) hippocampal interneurons
Source: Front Synaptic Neurosci. 2015 Feb 27;7:2. doi: 10.3389/fnsyn.2015.00002 (PMC4343010; doi:10.3389/fnsyn.2015.00002)
Supplement: Supplementary file 1 [file DataSheet1.DOCX]

***Supplementary Material***

**Dendritic distributions of *I*h channels in experimentally-derived multi-compartment models of oriens-lacunosum/moleculare (O-LM) hippocampal interneurons**

**Vladislav Sekulić1,2*, Tse-Chiang Chen1#, J Josh Lawrence4,5, Frances K Skinner1,3,2**

1 Toronto Western Research Institute, University Health Network, Toronto, Toronto, Ontario, M5T 2S8, Canada

2 Department of Physiology, University of Toronto, Toronto, Ontario, M5S 2J7, Canada

3 Department of Medicine (Neurology), University of Toronto, Toronto, Ontario, M5S 2J7, Canada

4 Center for Structural and Functional Neuroscience, University of Montana, Missoula, Montana, 59812, USA

5 Department of Biomedical and Pharmaceutical Sciences, University of Montana, Missoula, Montana, 59812, USA

# Current address: Department of Medical Biophysics, University of Toronto, Toronto, Ontario M5S 2J7, Canada

*** Correspondence:** Vladislav Sekulić, Toronto Western Research Institute, University Health Network, Toronto, Ontario, M5T 2S8, Canada. vlad.sekulic@utoronto.ca

**Keywords: h-channels, Ih, dendrites, hippocampus, interneurons, multi-compartment model.**

1. **Time constant equation parameters**

The time constant of activation for *I*_h_ used in this work follows the general form of,

$$\tau= \frac{t_{1}}{t_{2}e^{t_{3}V+t_{4}}+ t_{5}e^{t_{6}V+t_{7}}}+ t_{8}$$

The default values of *t*_1_, …, *t*_8_ for the model are the same as the parameters of the activation time constant used by Saraga et al. (2003), who used a modified version of Huguenard & McCormick (1992) to fit the two data points of Maccaferri & McBain (1996). In optimizing these parameter values, they were given the following constraints: *t*_1_ ≥ 0, *t*_2_ ≥ 0, *t*_3_ ≤ 0, *t*_4_ ≤ 0, *t*_5_ ≥ 0, *t*_6_ ≥ 0; *t*_7_ and *t*_8_ were allowed to take on any negative or positive value. Given this, the minimum value of τ is *t*_8_. The default values of *t*_1_, …, *t*_8_ are given in Supplementary Table 1, below.

Each parameter affects the time constant τ as follows. *t*_1_ scales the time constant linearly. *t*_2_, *t*_3_, *t*_4_ affect the left or more negative side of the time constant curve while *t*_5_, *t*_6_, *t*_7_ affect the right or more positive side of the time constant. *t*_8_ is the offset of the entire time constant.

Looking at the reciprocal picture of the time constant, we get a “bowl” as made by the sum of two exponentials, subject to the constraints.​ An increase in *t*_2_ means an increase of the left side of the bowl due to vertical scaling of the left-sided exponential or the left side of the bowl, and a decrease in *t*_2_ means a decrease in vertical scaling (as shown in the *t*_2_ graph). To see its effects on the time constant means to take the reciprocal of the effects on the "bowl"; namely, increase in *t*_2_ means the left side of the time constant is lower while a decrease in *t*_2_ means the left side is higher. The right side is relatively unaffected, as shown.

Again, using the bowl metaphor, *t*_3_ affects the horizontal scaling or scaling of the voltage. An increase in magnitude of *t*_3_ results in the horizontal compression of the left side of the bowl. This works the same way for the time constant, as taking a reciprocal does not affect the horizontal scaling. This is shown in the *t*_3_ variation graph.

*t*_4_ affects the placement or horizontal offset of the left side of the bowl. The more negative the *t*_4_ value, the farther out into the negative values the left side of the bowl is. This means that the bowl is flatter and wider in the center, where its values are lower or closer to 0. Taking the reciprocal of the bowl, we can see that this means that the time constant will be larger and wider, skewed towards the left (more negative) side.

The same arguments hold for *t*_5_, *t*_6_, and *t*_7_ where the effects are felt on the right or more positive side of the bowl and thus, time constant. The analogous pairs are *t*_2_ and *t*_5_, *t*_3_ and *t*_6_, *t*_4_ and *t*_7_.

The effect of each parameter is shown visually in Supplementary Figures 1-8 (below). It is important to note that the effects of these parameters are not independent. This can be noted visually from the graphs as well as the above descriptions. What can be clearly stated is that with the parameterization and constraints, *t*_2_, *t*_3_, and *t*_4_ affect the left side of the time constant curve and *t*_5_, *t*_6_, and *t*_7_ affect the right side of the time constant curve. This can be mathematically appreciated by noting that the above time constant expression can be rewritten as:

$$\tau= \frac{1}{ae^{t_{3}V}+ be^{t_{6}V}}+ t_{8}$$

where,

$$a=\frac{t_{2}}{t_{1}}e^{t_{4}}, b=\frac{t_{5}}{t_{1}}e^{t_{7}}$$

Thus, there are 5 independent parameters: *t*_3_, *t*_6_, *t*_8_, *a*, and *b*.

1. **Supplementary Figures and Tables**

The default values of the time constant of activation for *I*_h_ from Saraga et al. (2003) are given in Supplementary Table 1.

## Supplementary Tables

**Supplementary Table 1. Default values of equation for the time constant of activation of *I*_h_.**

| **Original parameter** | **Value** |
| --- | --- |
| ***t*_1o_** | 1 |
| ***t*_2o_** | 1 |
| ***t*_3o_** | -0.116 |
| ***t*_4o_** | -17.9 |
| ***t*_5o_** | 1 |
| ***t*_6o_** | 0.09 |
| ***t*_7o_** | -1.84 |
| ***t*_8o_** | 100 |

## Supplementary Figures


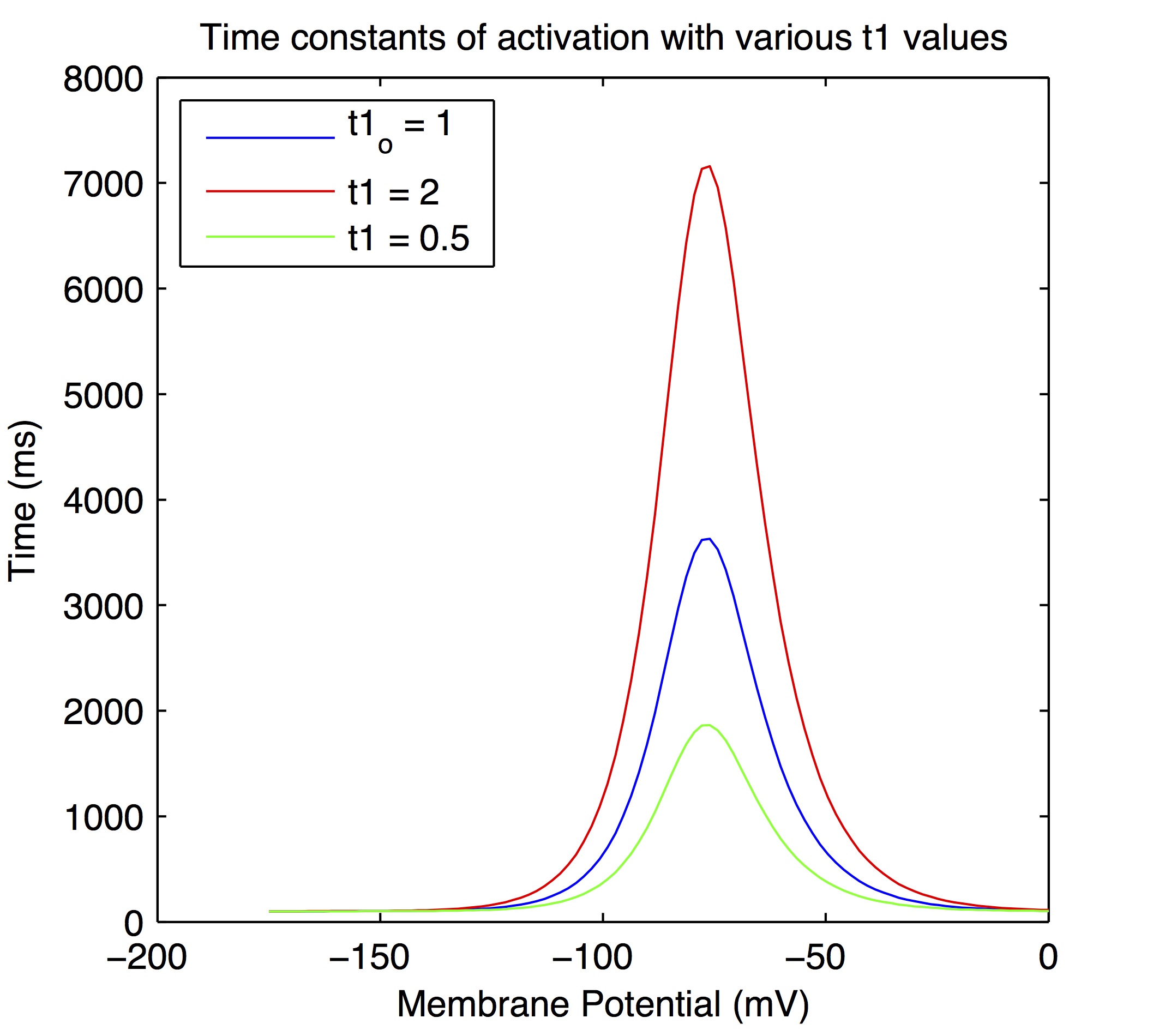


Supplementary Figure 1. Effects of changing the *t*_1_ parameter on the shape of the *I*_h_ activation time constant. The *t*_1o_ curve refers to the original model of Saraga et al. (2003) modeled after the data in Maccaferri and McBain (1996).


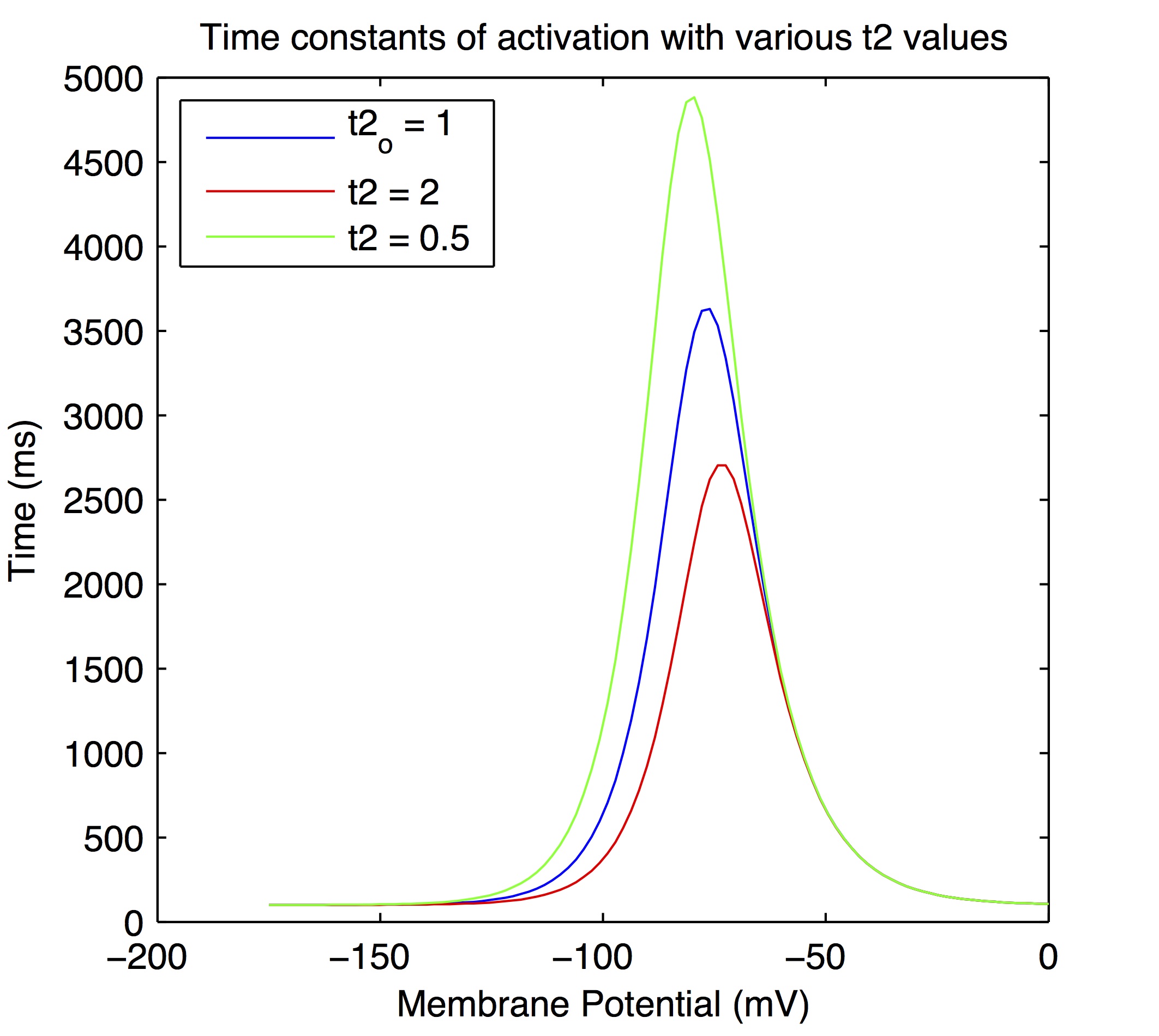


Supplementary Figure 2. Effects of changing the *t*_2_ parameter on the shape of the *I*_h_ activation time constant. The *t*_2o_ curve refers to the original model of Saraga et al. (2003) modeled after the data in Maccaferri and McBain (1996).


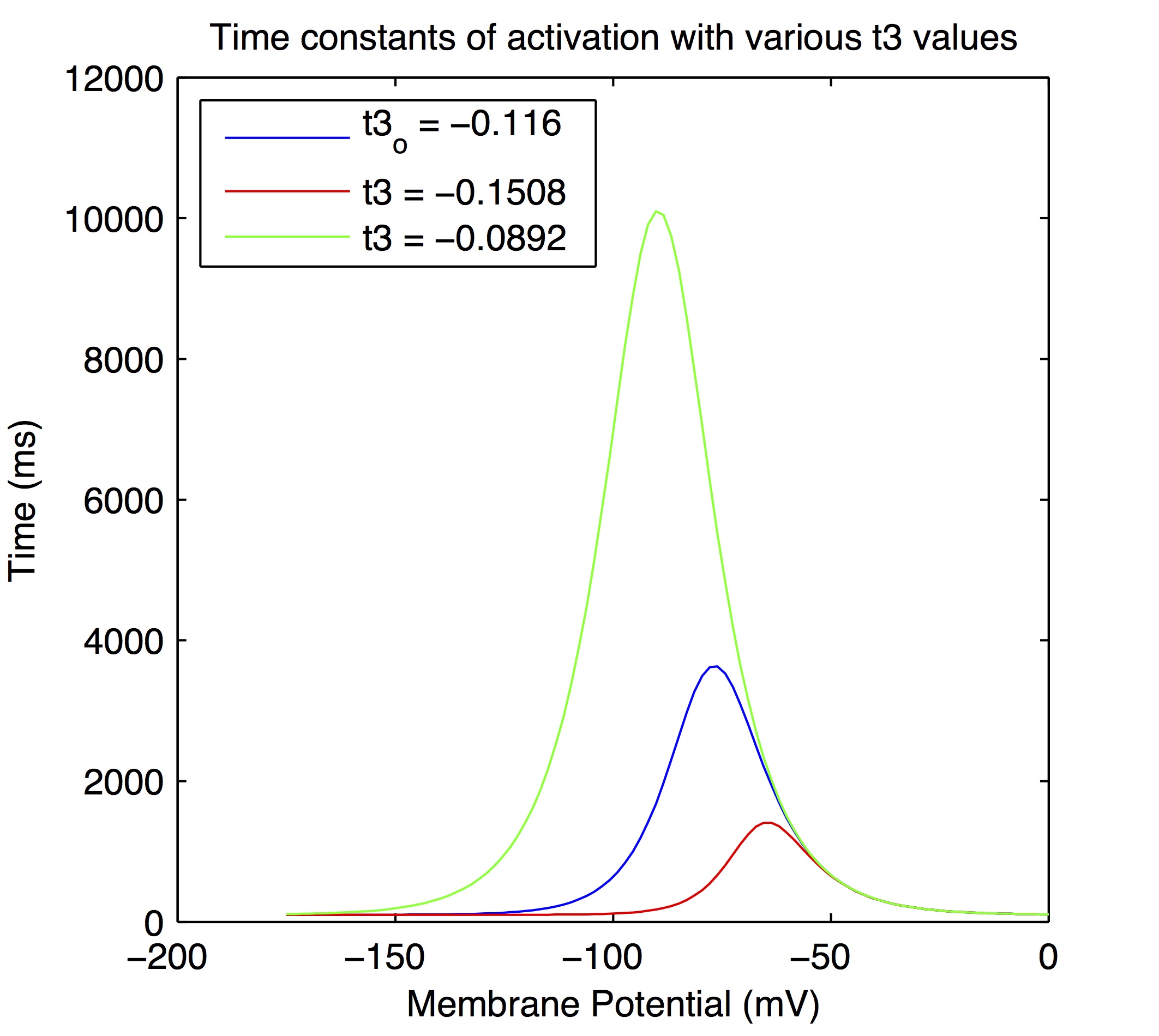


Supplementary Figure 3. Effects of changing the *t*_3_ parameter on the shape of the *I*_h_ activation time constant. The *t*_3o_ curve refers to the original model of Saraga et al. (2003) modeled after the data in Maccaferri and McBain (1996).


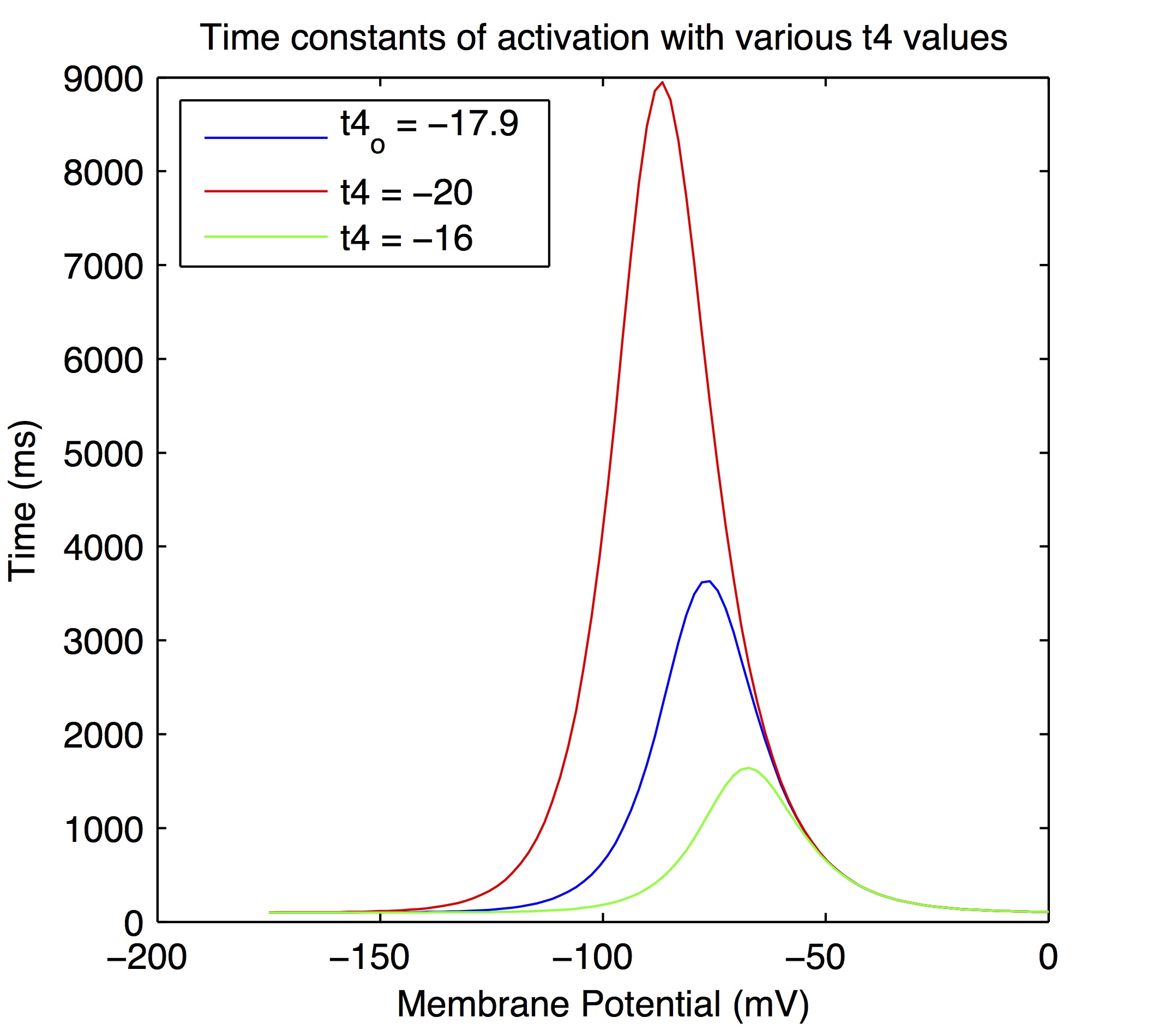


Supplementary Figure 4. Effects of changing the *t*_4_ parameter on the shape of the *I*_h_ activation time constant. The *t*_4o_ curve refers to the original model of Saraga et al. (2003) modeled after the data in Maccaferri and McBain (1996).


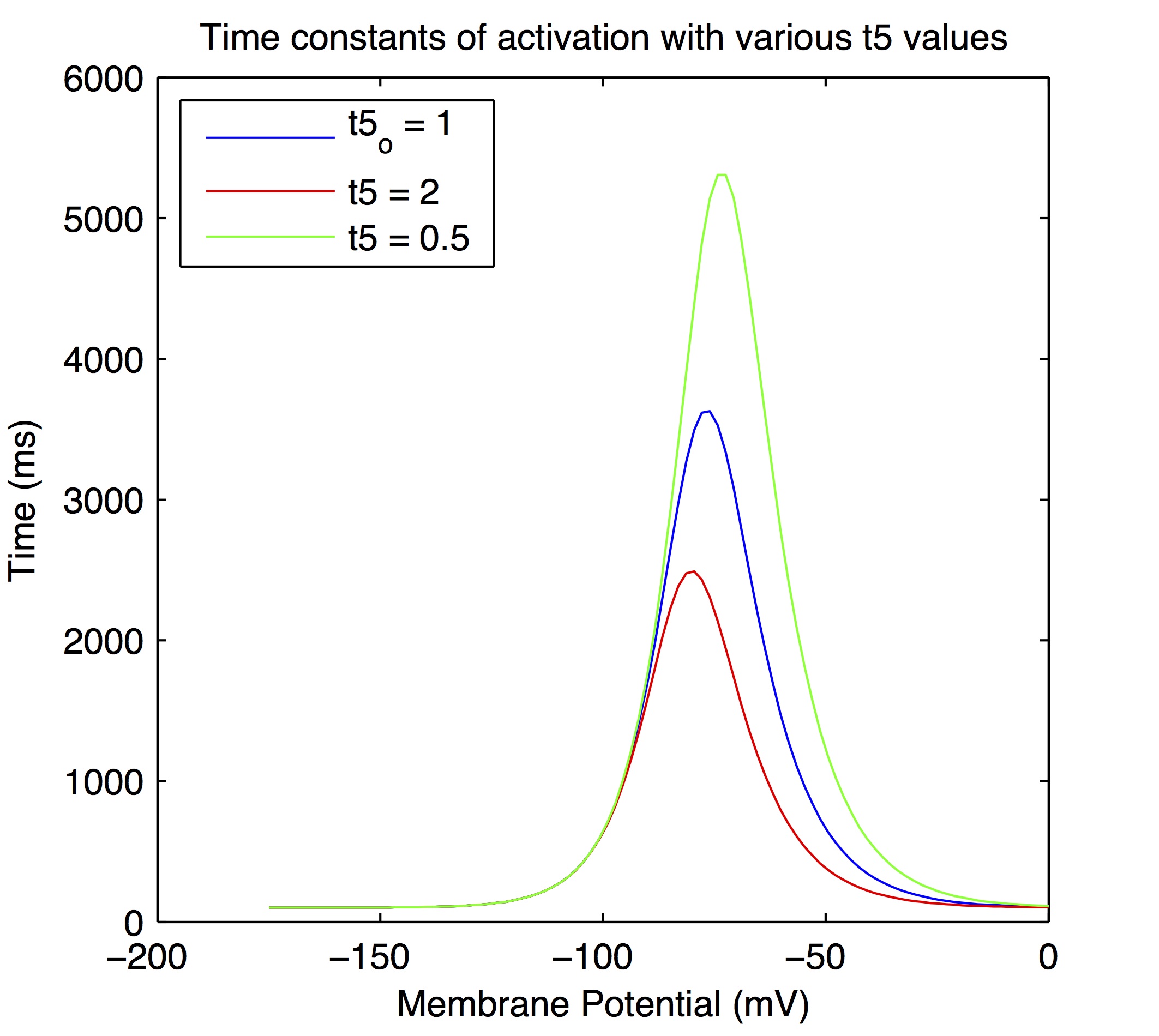


Supplementary Figure 5. Effects of changing the *t*_5_ parameter on the shape of the *I*_h_ activation time constant. The *t*_5o_ curve refers to the original model of Saraga et al. (2003) modeled after the data in Maccaferri and McBain (1996).


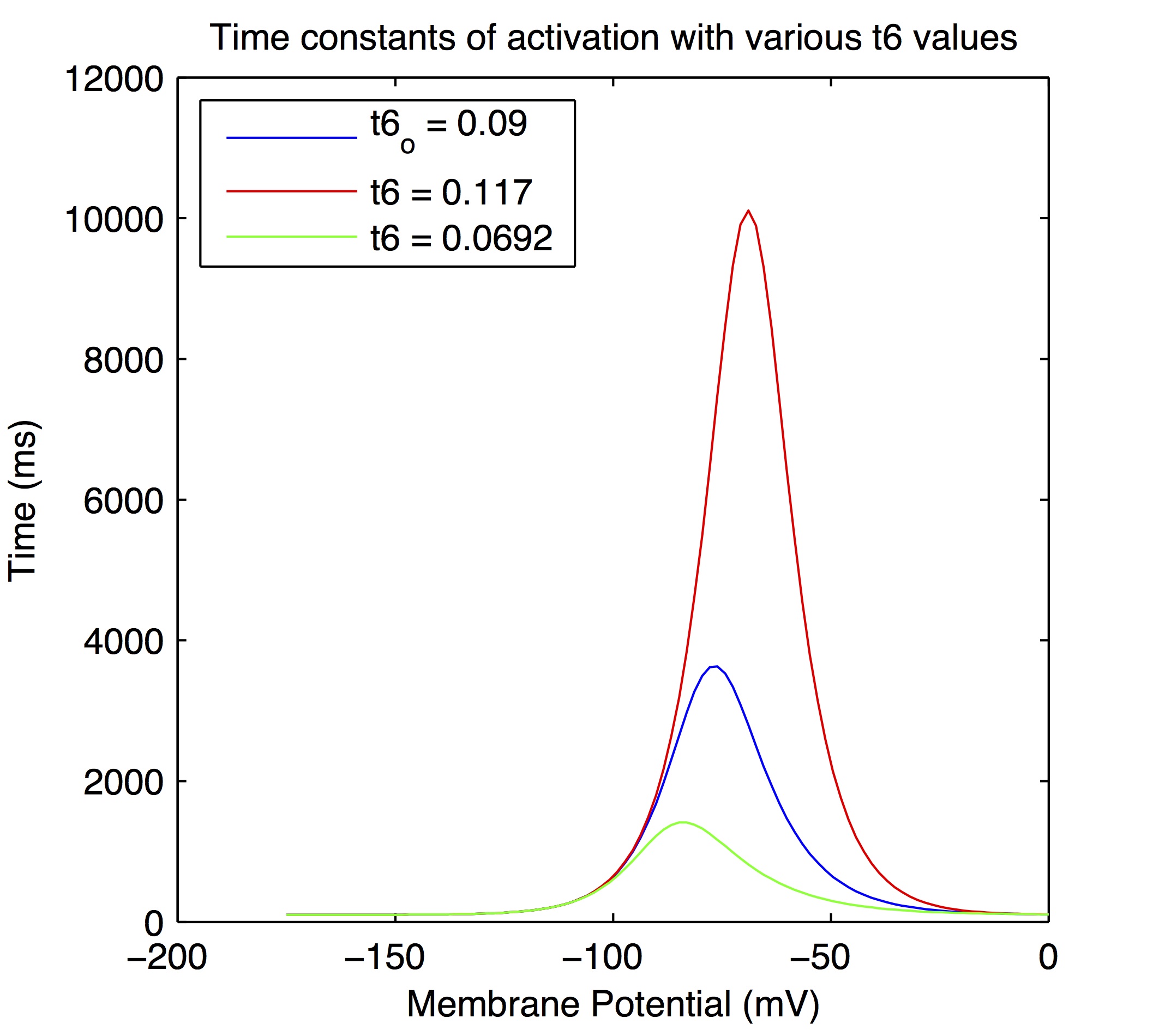


Supplementary Figure 6. Effects of changing the *t*_6_ parameter on the shape of the *I*_h_ activation time constant. The *t*_6o_ curve refers to the original model of Saraga et al. (2003) modeled after the data in Maccaferri and McBain (1996).


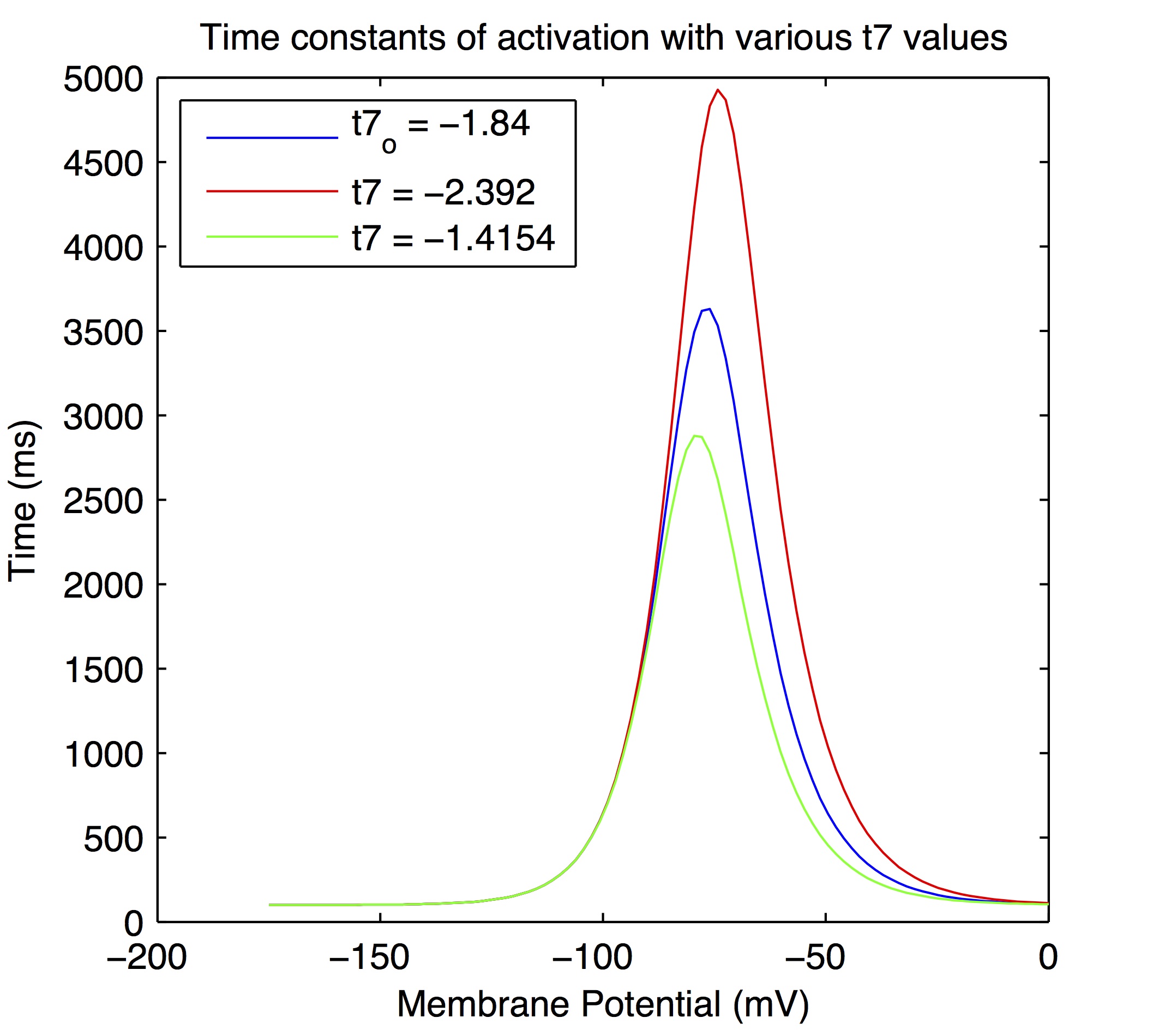


Supplementary Figure 7. Effects of changing the *t*_7_ parameter on the shape of the *I*_h_ activation time constant. The *t*_7o_ curve refers to the original model of Saraga et al. (2003) modeled after the data in Maccaferri and McBain (1996).


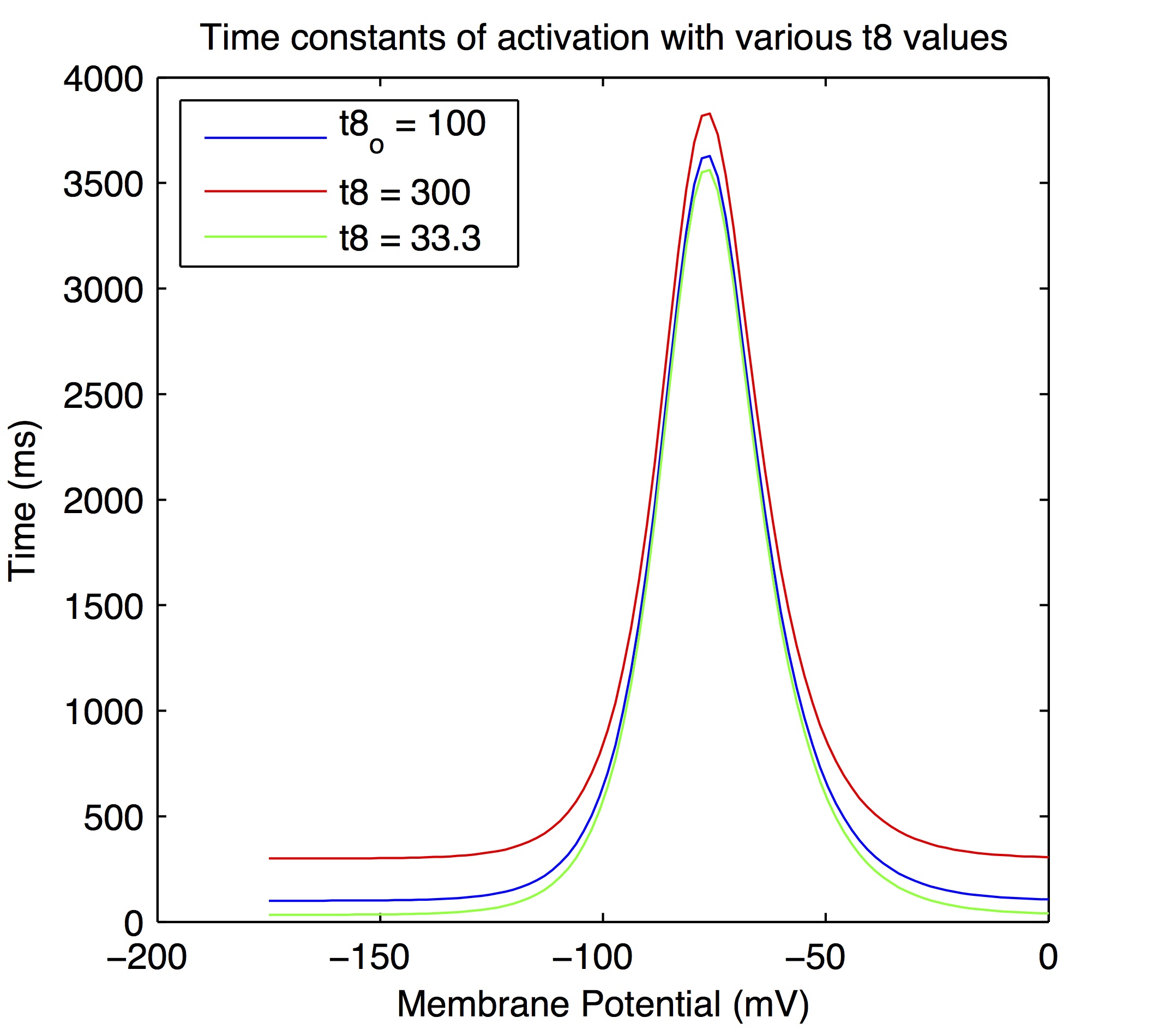


Supplementary Figure 8. Effects of changing the *t*_8_ parameter on the shape of the *I*_h_ activation time constant. The *t*_8o_ curve refers to the original model of Saraga et al. (2003) modeled after the data in Maccaferri and McBain (1996).

1. **References**

Huguenard, J. R., and McCormick, D. A. (1992). Simulation of the currents involved in rhythmic oscillations in thalamic relay neurons. *J Neurophysiol*, 68, 1373-1383.

Saraga, F., Wu, C.P., Zhang, L., & Skinner, F. K. (2003). Active dendrites and spike propagation in

multi-compartment models of oriens-lacunosum/moleculare hippocampal interneurons. *The Journal of Physiology*, 552(3), 673-689. doi:10.1113/jphysiol.2003.046177

Maccaferri, G., & McBain, C. (1996). The hyperpolarization-activated current (Ih) and its contribution to pacemaker activity in rat CAI hippocampal stratum oriens-alveus interneurones. *Journal of Physiology*, 497(1), 119-130.
